# Proteomic analysis uncovers common effects of IFN-γ and IL-27 on the HLA class I antigen presentation machinery in human cancer cells

**DOI:** 10.18632/oncotarget.12235

**Published:** 2016-09-24

**Authors:** Andrea Petretto, Grazia Carbotti, Elvira Inglese, Chiara Lavarello, Maria Pia Pistillo, Valentina Rigo, Michela Croce, Luca Longo, Stefania Martini, Paola Vacca, Silvano Ferrini, Marina Fabbi

**Affiliations:** ^1^ Core Facilities-Proteomics Laboratory, Istituto Giannina Gaslini, Genoa, Italy; ^2^ Department of Integrated Oncological Therapies, IRCCS AOU San Martino-IST Istituto Nazionale per la Ricerca sul Cancro, Genoa, Italy; ^3^ Tumor Epigenetics Unit, IRCCS AOU San Martino-IST Istituto Nazionale per la Ricerca sul Cancro, Genoa, Italy; ^4^ Department of Experimental Medicine (DIMES), University of Genoa, Genoa, Italy

**Keywords:** IL-27, IFN-γ, proteomic analysis, HLA class I, cancer cells, Immunology and Microbiology Section, Immune response, Immunity

## Abstract

IL-27, a member of the IL-12-family of cytokines, has shown anti-tumor activity in several pre-clinical models due to anti-proliferative, anti-angiogenic and immune-enhancing effects. On the other hand, IL-27 demonstrated immune regulatory activities and inhibition of auto-immunity in mouse models. Also, we reported that IL-27, similar to IFN-γ, induces the expression of IL-18BP, IDO and PD-L1 immune regulatory molecules in human cancer cells. Here, a proteomic analysis reveals that IL-27 and IFN-γ display a broad overlap of functions on human ovarian cancer cells. Indeed, among 990 proteins modulated by either cytokine treatment in SKOV3 cells, 814 showed a concordant modulation by both cytokines, while a smaller number (176) were differentially modulated. The most up-regulated proteins were common to both IFN-γ and IL-27. In addition, functional analysis of IL-27-regulated protein networks highlighted pathways of interferon signaling and regulation, antigen presentation, protection from natural killer cell-mediated cytotoxicity, regulation of protein polyubiquitination and proteasome, aminoacid catabolism and regulation of viral protein levels.

Importantly, we found that IL-27 induced HLA class I molecule expression in human cancer cells of different histotypes, including tumor cells showing very low expression. IL-27 failed only in a cancer cell line bearing a homozygous deletion in the *B2M* gene. Altogether, these data point out to a broad set of activities shared by IL-27 and IFN-γ, which are dependent on the common activation of the STAT1 pathway. These data add further explanation to the anti-tumor activity of IL-27 and also to its dual role in immune regulation.

## INTRODUCTION

IL-27 is a heterodimeric cytokine, consisting of p28 (IL-27A) and EBV-induced gene 3 (EBI3) chains, which belongs to a cytokine family comprising IL-12, IL-23, IL-30 and IL-35 [1-3]. The primary sources of IL-27 are macrophages and dendritic cells activated by TLR stimulation [4-6]. IL-27 binds to a receptor formed by gp130 and WSX1/IL-27RA chains, which signals through the STAT1/3 pathway. It mediates pleiotropic effects on T, B and NK lymphocytes, macrophages, dendritic cells and non-hematopoietic cells, and also on different types of normal and neoplastic cells [1-3].

The effects of IL-27 on the immune system are dual, as it may have immune enhancing or immune regulatory properties, in relationship to the biological context [7]. On one hand, IL-27 induces T-bet expression and Th1 cell differentiation [8] and supports the generation of effector CD8^+^ T cells expressing granzyme B [9]. Also, it has been proposed that IL-27 enhances the survival of anti-tumor CTLs and induces a peculiar stem cell-like Tc1 effector phenotype, characterized by the expression of T-bet, Eomes, Bcl1, Sca1 and IL-10 [10].

On the other hand, IL-27 has anti-inflammatory and immune regulatory functions, which may limit the development of auto-immune or -inflammatory diseases [11-13]. Indeed, IL-27 mediates the induction of IL-10-producing type-1 Treg cells [14] and expression of PD-L1 in naïve T cells, which convert into Treg cells and protect mice from autoimmune encephalomyelitis [13]. Also, IL-27 produced by DCs upon recognition of apoptotic tumor cells induces expression of immune-suppressive CD39 ecto-ATPase on Treg cells, which inhibits CTL responses [15].

Notwithstanding these immune regulatory effects, IL-27 has shown anti-tumor activity in several cancer models *in vitro* and *in vivo*, not only through the activation of anti-tumor immune responses but also through direct effects on the tumor cells. For example, IL-27 inhibits angiogenesis and neoplastic cell proliferation in different hematologic neoplasia including acute myeloid leukemia [16], acute B lymphoblastic leukemia [17] and myeloma [18]. In addition, IL-27 suppressed the growth of prostate cancer cells *in vitro* and in xenograft models, where it also exerted anti-angiogenic properties [19]. Similarly, it limited tumor growth and angiogenesis through the induction of anti-angiogenic chemokines in a syngeneic mouse melanoma model [20]. Recent findings indicated that IL-27 suppresses the expression of stem cell and mesenchymal transition genes in lung cancer cells [21]. Altogether immune-stimulatory activities and direct anti-tumor effects support the possible usage of IL-27 for tumor therapy.

However, our recent data showed that, beyond these anti-tumor effects, IL-27 also induces the expression of immune regulatory molecules such as IL-18BP, the natural inhibitor of the Th1-inducing cytokine IL-18, in ovarian cancer cells [22]. Perhaps more importantly, it induced the expression of the immune-suppressive molecules IDO and PD-L1, in human cancer cells, through the activation of STAT1 or STAT3 pathways, respectively [23]. It is noteworthy that both IL-27 and IFN-γ induce IL-18BP, PD-L1, and IDO, suggesting that these cytokines may have other, yet unknown, common effects. Indeed, the activation of STAT1 tyrosine phosphorylation (P-Tyr701) by both cytokines supports the hypothesis that they may activate a partially overlapping genetic program. However, IL-27, but not IFN-γ activates STAT3 tyrosine phosphorylation, which may trigger IL-27-specific effects [2].

To better dissect the effects of IL-27 and IFN-γ on ovarian cancer cells, we used a proteomic approach to identify the profile of cytokine-regulated proteins. Our present data indicate that IL-27 and IFN-γ concordantly modulated a broadly overlapping set of proteins including intracellular mediators of IFN signaling, antigen presentation machinery components and antiviral proteins. Only a small set of proteins was specifically regulated by each cytokine.

## RESULTS

### Proteomic analysis of IFN-γ- and IL-27-regulated proteins in ovarian cancer cell lines reveals a large set of common effects

To gain more information on IL-27 effects on cancer cells, we used a proteomic approach based on high-resolution mass spectrometry on cell lysates from cytokine-treated or untreated cells, in triplicate independent experiments. We initially chose the SKOV3 ovarian cancer cell line, which has been widely utilized as a serous ovarian adenocarcinoma cell model, and responds to IL-27 stimulation by up-regulating the expression of immune regulatory IL-18BP, IDO, and PD-L1 molecules [22, 23]. Since also IFN-γ up-regulates these molecules, we compared IL-27 and IFN-γ effects on the proteome.

Data processing through the MaxQuant software identified a total of 6582 proteins, of which 5610 were quantified using a Label-Free Quantitation approach. Quantitation requires that a protein is identified in all three biological replicates at least in one treatment condition.

**Figure 1:**
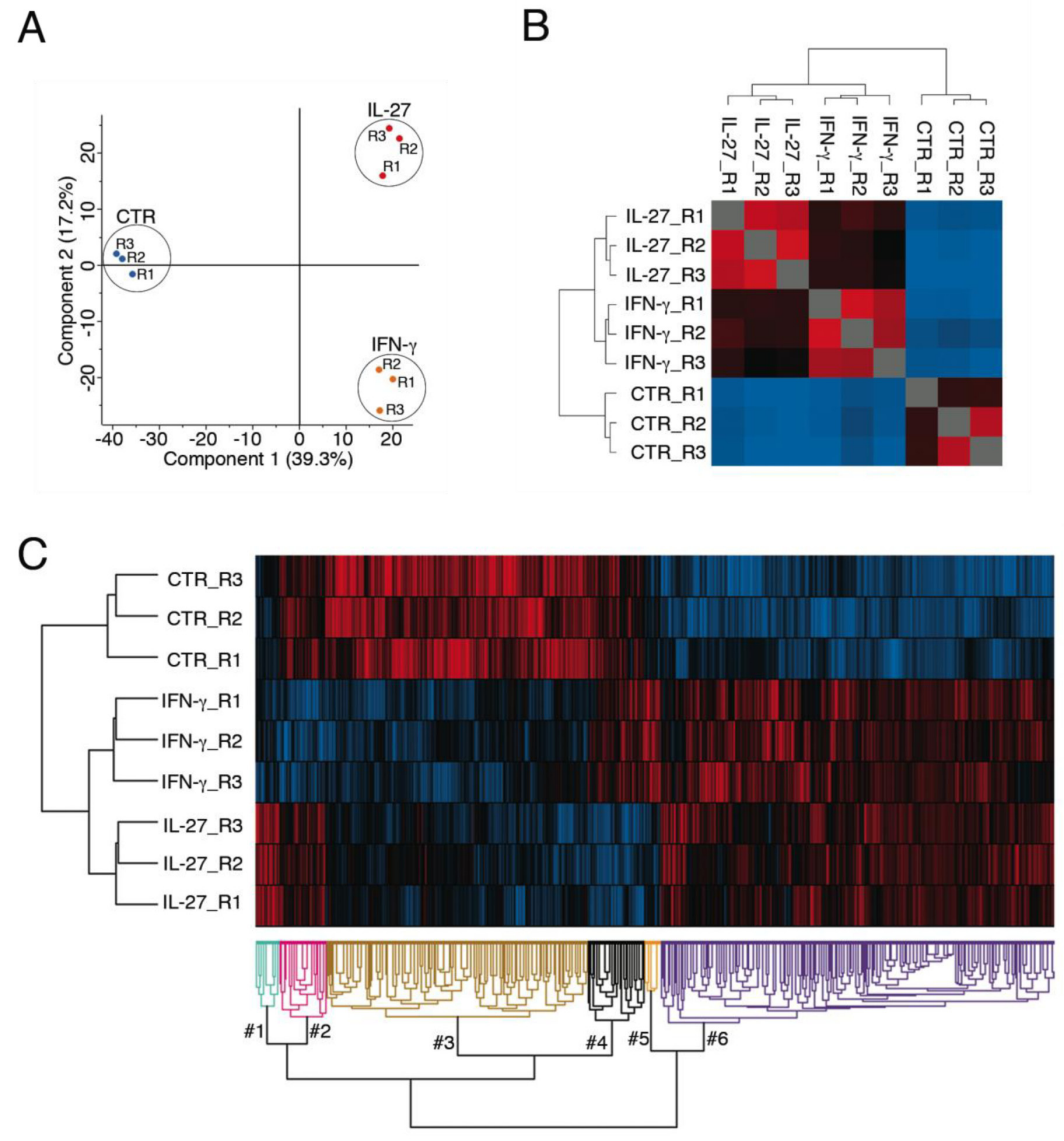
Principal component analysis, Pearson's correlogram and unsupervised hierarchical clustering analysis of untreated, IFN-γ-or IL-27-treated SKOV3 cells **A.** Two-dimensional scatter plot of the principal component analysis of SKOV3 Untreated (blue), IFN-γ- (orange) and IL-27-treated (red dots) samples.**B.** The Pearson's correlogram depicts the coefficient values in a pseudo-color scale, which extends from 0.1 (light blue) to 0.9 (red). The dendrogram displays the results of an unsupervised hierarchical-clustering analysis placing similar Pearson's coefficient values near each other. All the samples cluster according to treatment.**C.** Unsupervised hierarchical-clustered heatmap of 990 proteins identified by Multiple-samples test ANOVA performed on the SKOV3 cell line. The amount of each protein in individual samples is represented by the color scheme in which red and blue indicate high and low expression of proteins, respectively. Three independent biological replicates of cells treated with IL-27 or IFN-γ or left untreated (CTR) are shown. Proteins are clustered into 6 groups according to their expression value. Cluster #3 and cluster #6 represent a concordant modulation by IFN-γ and IL-27 treatments. In particular, 489 were up-regulated in cluster #6 and 325 were down-regulated in cluster #3 by both cytokines. A smaller number of proteins (176) was differentially modulated by the two cytokines and clustered into 4 small groups, relative to untreated cells.

Principal-component and hierarchical-clustering analyses of untreated, IFN-γ- or IL-27-treated replicates were performed to highlight any similarities or differences among the three groups. The two-dimensional scatter plot of the principal components shows that proteins from the different SKOV3 samples underwent a good separation according to treatments (Figure 1A). The same result was obtained using Pearson's correlogram associated with hierarchical-clustering analysis, based on the abundance of proteome profile (Figure 1B). Interestingly, average Pearson's coefficient (0.96) was very close between the IFN-γ- and IL-27-treated samples suggestive of broadly overlapping effects of the two cytokines. Furthermore, multiple-samples test ANOVA and unsupervised hierarchical-clustered heatmap showed that among 990 proteins modulated by either cytokine treatment, 814 showed a concordant modulation (Figure 1C). In particular, 489 were up-regulated, and 325 were down-regulated by both cytokines. On the other hand, a smaller number of proteins (176) were differentially modulated by the two cytokines, relative to untreated cells.

**Figure 2:**
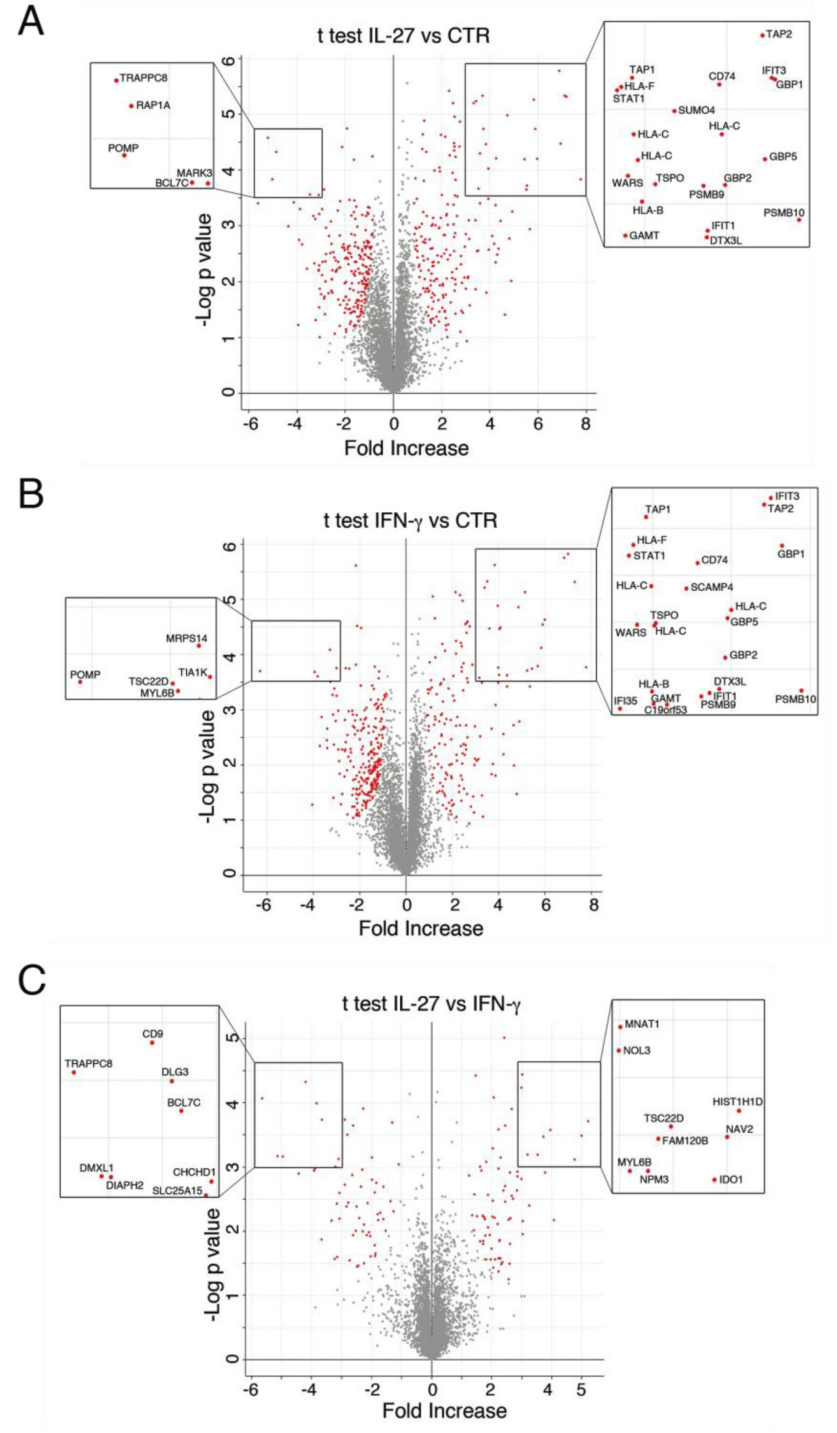
Volcano plot representation of differentially expressed proteins Plots represent untreated (CTR) and IL-27-treated **A.**, CTR and INF-γ-treated **B.** and IL-27 and IFN-γ-treated **C.** SKOV3 cell samples. Red dots represent proteins that display both large-magnitude fold-changes (x-axis, to the right there are proteins up-regulated after treatment) as well as high statistical significance (−log10 of P value, y-axis). The black line shows where FDR = 0.05 and s0 = 0.5. Gray dots represent proteins whose fold-change is < 2 (log2 = 1) or the *P* > 0.05. Proteins labeled with gene name (inset) are the most modulated ones.

A Student's *T*-test analysis was performed to characterize the differences and the reciprocal relationships between groups of treatments and graphically represented by Volcano plots (Figure 2). Figure 2A shows 412 differentially expressed proteins (196 up- and 216 down-regulated) by IL-27-treatment. IFN-γ modulated the expression of 406 proteins of which 161 up- and 245 down-regulated (Figure 2B). Strikingly, the most up-regulated proteins (Fold increase > 3 and *P* < 0.0005, as highlighted in the figure inset) were common to both IFN-γ and IL-27. These included some well-known intracellular mediators of IFN activities including STAT1, Interferon-Induced Protein With Tetratricopeptide Repeats (IFIT) 1 and 3, Guanylate Binding Protein Interferon-Inducible (GBP)1, 2 and 5, and the enzymes Guanidinoacetate N-methyltransferase (GAMT) and Tryptophanyl-tRNA synthetase (WARS). Importantly, a large group of strongly up-regulated proteins belongs to the proteasome or the HLA class I antigen presentation machinery (APM). These included Deltex 3 Like E3 Ubiquitin Ligase (DTX3L), proteasome subunit beta (PSMB) 9 and 10, Transporter ATP-binding cassette (TAP) 1 and 2 and HLA-B, -C and -F heavy chains. In addition, CD74, the HLA-DR antigens-associated invariant chain, was one of the most up-regulated proteins.

Among the most down-regulated proteins the Proteasome Maturation Protein (POMP) was diminished by both cytokines, possibly due to degradation, which occurs before the maturation of the 20S proteasome is complete.

A similar analysis was performed in another IL-27-responsive ovarian cancer cell line named OC316 [24], which showed a similar pattern of proteins, commonly modulated by IL-27 and IFN-γ (Supplementary Figure S1 and S2). In this cell line, the IFN-γ-inducible Caspase-1 (CASP1) [25] was among the most IL-27 up-regulated proteins.

A comparison between IL-27 and IFN-γ showed that only 132 proteins were differentially modulated in SKOV3 cells, of which 60 were more expressed in cells treated with IFN-γ and 72 in IL-27-treated cells, indicating that besides extensive common effects the two cytokines also display specific ones (Figure 2C). Intriguingly, the set of proteins specifically modulated by IL-27 differed between SKOV3 and OC316 (Supplementary Figure S2C), suggesting that the IL-27-specific effects may be different according to the cell type.

### Bioinformatics analyses of IL-27-regulated proteins

**Figure 3:**
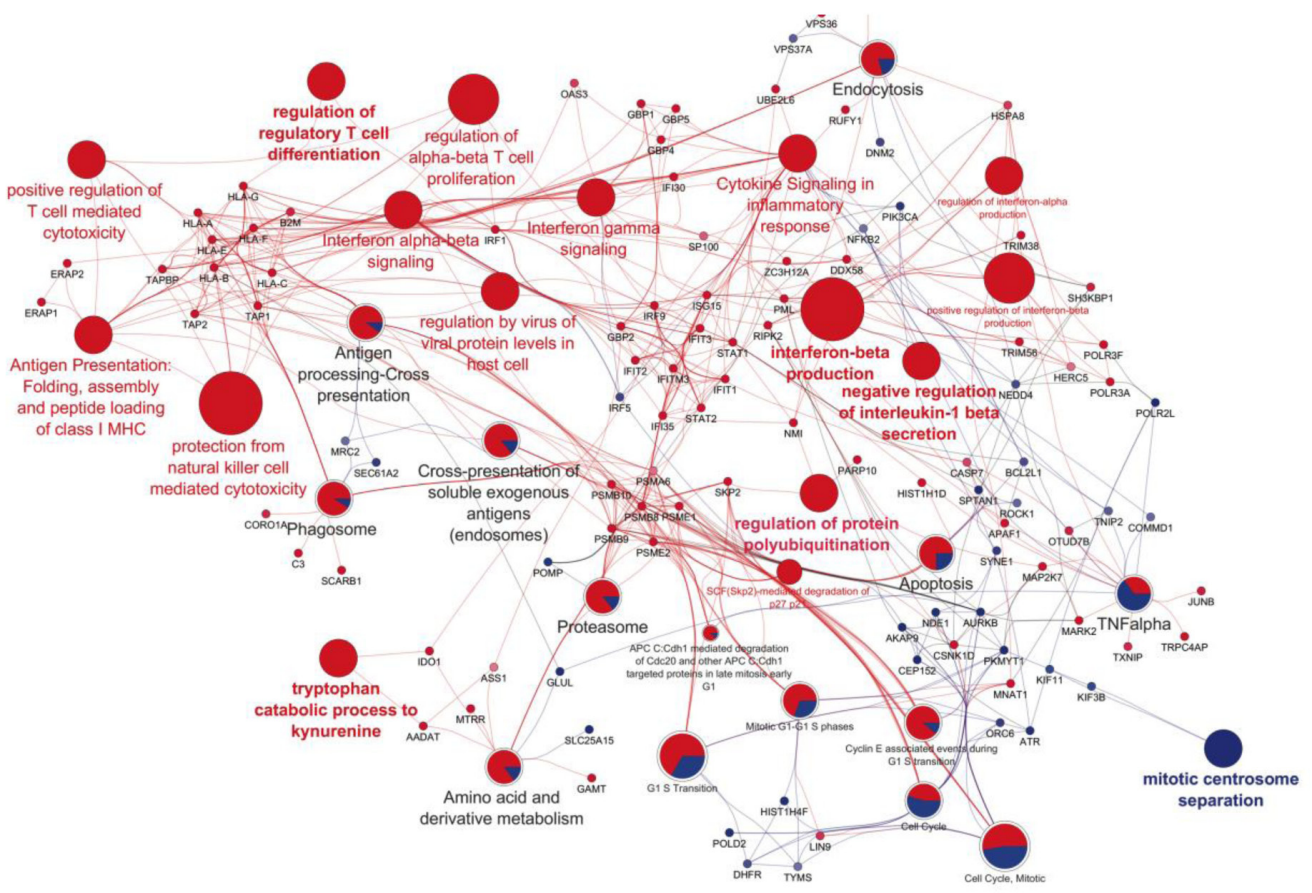
Functional networks of IL-27-modulated proteins in SKOV3 cells The network was obtained from proteins showing significant (*t*-test) variations between IL-27 treatment and control. The network was built through the merge between a first network obtained according to ClueGO setup and a second one built by GENEMANIA app. Each big node represents a Gene Ontology Biological Process term or a Reactome pathway, while the small node represents query proteins used for the analysis from which previous terms are obtained. The node size represents the P value obtained from two-side hypergeometric test corrected by the Bonferroni step-down method. The protein expression is represented by the color scheme in which red and blue indicate high and low expression of proteins. The same colors are used for the node terms in which the pie represents the percentage of protein up or down regulated.

To gain further information on the biological effects of IL-27, functional networks of IL-27-induced protein modulations were built according to the ClueGO and GENEMANIA setups. Among IL-27 up-regulated protein networks and processes we found: i) Interferon signaling and regulation, ii) antigen presentation, folding assembly and peptide loading on MHC class I, iii) protection from natural killer cell-mediated cytotoxicity, iv) regulation of protein polyubiquitination and proteasome, v) aminoacid metabolism and tryptophan catabolism and vi) regulation of viral protein levels (Figure 3). Altogether these data point out to a large set of pathways mostly shared with interferon activities. In particular, IL-27 up-regulated the expression of several proteins involved in IFN signaling and anti-viral activities, such as STAT1, the Interferon Response Factor (IRF)-1 and 9 [26], IFIT1, 2, and 3 [27] and the GBP-1, −2, −4 and −5 [28]. GBP-1 is one of the most significantly induced proteins in cells exposed to IFN and is involved in its antibacterial, antiviral [29], anti-angiogenic [30, 31] and anti-tumor activities [32]. In addition, IL-27 up-regulated the proteins of the MHC-class I APM including TAP-1 and −2, TAP-binding protein (TAPBP), HLA-A, -B, -C, -E, -G and -F heavy chains, β2-microglobulin (B2M), Endoplasmic Reticulum Aminopeptidase (ERAP)-1 and −2 and several proteasome components, in SKOV3 cells. Similar findings, especially concerning the MHC class-I APM, were obtained with the OC316 cells (Supplementary Figure S3), with the notable exception of B2M, which was undetectable in this cell line. In OC316 cells, network analysis highlighted many nodes related to IFN anti-viral functions including negative regulation of viral process and genome replication. Indeed, several proteins up-regulated by both IL-27 and IFN-γ were anti-viral proteins, including IFIT1, 2 and 3 and the 2′-5′-OligoAdenylate Synthetase, (OAS)-1 and 3, the ubiquitin-like modifiers ISG15 and 20, and MX dynamin-like GTPase (MX)1.

### IL-27 inhibits cell proliferation and survival

**Figure 4:**
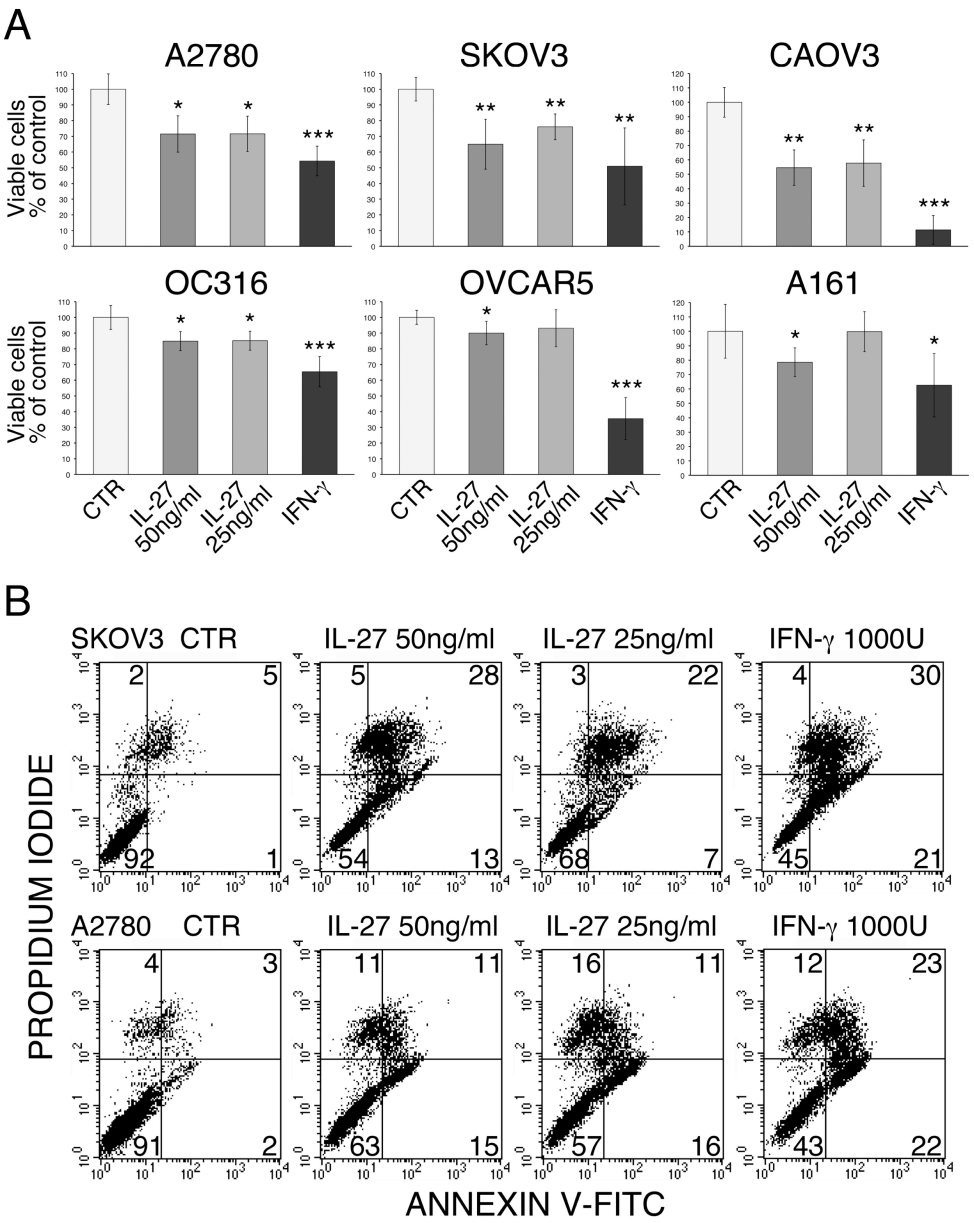
Anti-proliferative and pro-apoptotic effects of IL-27 on human ovarian cancer cell lines **A.** The proliferation and cell viability was evaluated using a standard MTT assay. Data are expressed as % of untreated control and are the mean±SD of six replicates. The experiment is representative of at least three independent ones showing consistent results. * 5E-5 < *P* < 0.05; **1E-7 < *P* < 5E-5; *** *P* < 1E-7. **B.** Apoptosis was measured by an Annexin V-FITC/PI staining and flow-cytometric analysis. Numbers indicate the % of cells in the relative quadrant (lower left: viable cells; lower right: early apoptotic; upper right: apoptotic; upper left: post-apoptotic cells). An experiment representative of three with consistent data is shown for two cell lines.

Since several of the commonly up-regulated proteins have been involved in anti-proliferative or pro-apoptotic effects of IFN-γ, we investigated whether IL-27 shares these functions on ovarian cancer cell lines. As shown in Figure 4A IL-27 inhibited cell proliferation, as detected by an MTT assay, although the OVCAR5 and OC316 cell lines were less sensitive to IL-27 inhibition than the other cell lines. We therefore investigated whether cell loss could be related to induction of apoptosis as detected by Annexin V-FITC/Propidium Iodide (PI) staining. Figure 4B and supplemental Figure S4 show that IL-27 at two different doses significantly increased the apoptotic cell death relative to untreated controls. In general, the anti-proliferative and pro-apoptotic effects of IL-27 were less pronounced than those of IFN-γ.

### IL-27 up-regulates surface HLA class I expression in cancer cells

**Figure 5:**
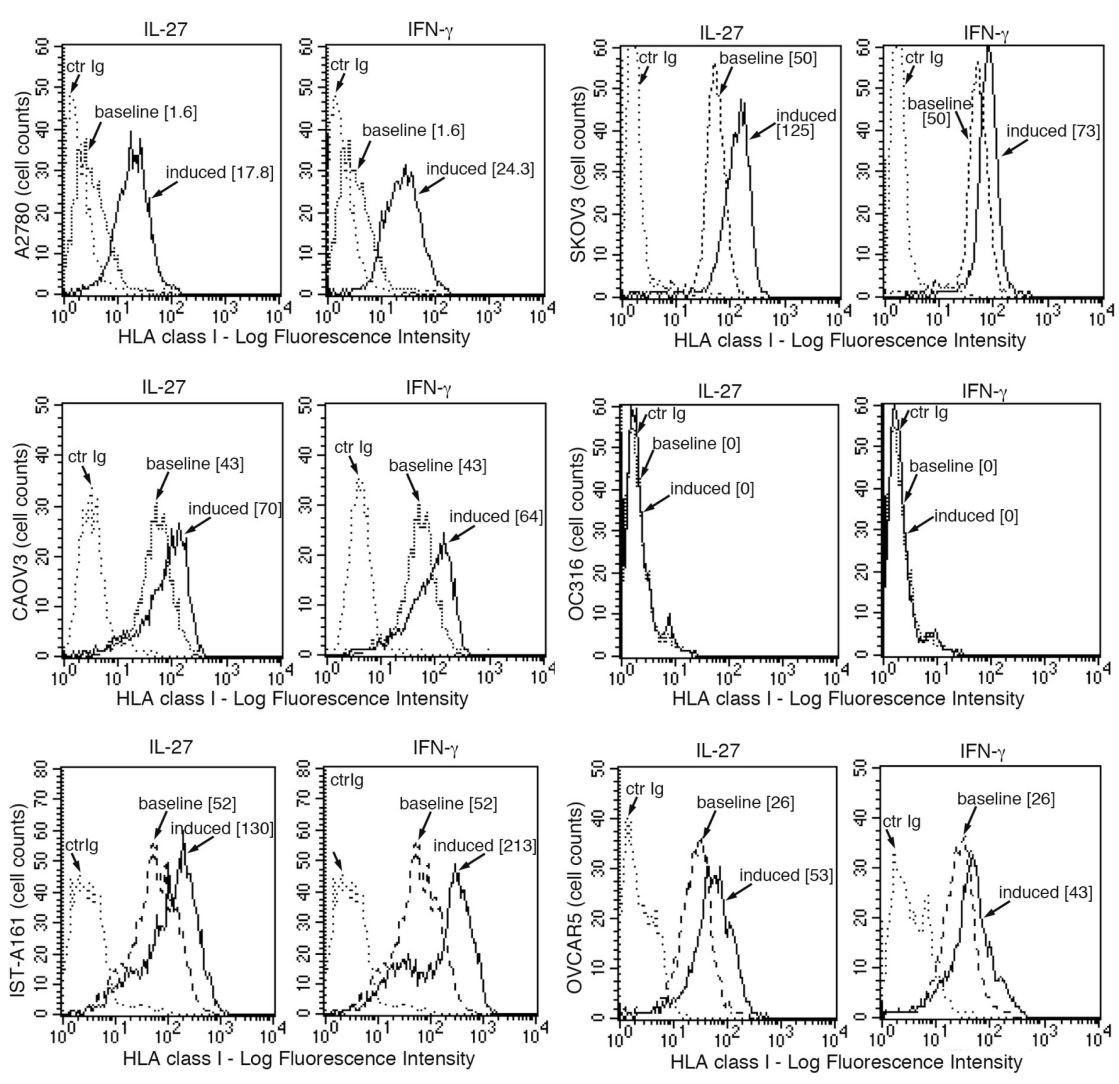
IL-27 increases HLA class I membrane expression in human ovarian cancer cells *in vitro* FACS analysis of surface HLA class I expression in five ovarian cancer cell lines and one primary culture (IST-A161, passage 15) of serous ovarian carcinoma cells from ascites, cultured in the presence of medium (baseline), IL-27 or IFN-γ (induced). Isotype-matched unrelated Ig staining control is indicated (ctrIg). Numbers in brackets are Median Fluorescence Intensity (MFI) values calculated as median HLA class I (W6/32 mAb) minus median Ig control.

**Figure 6:**
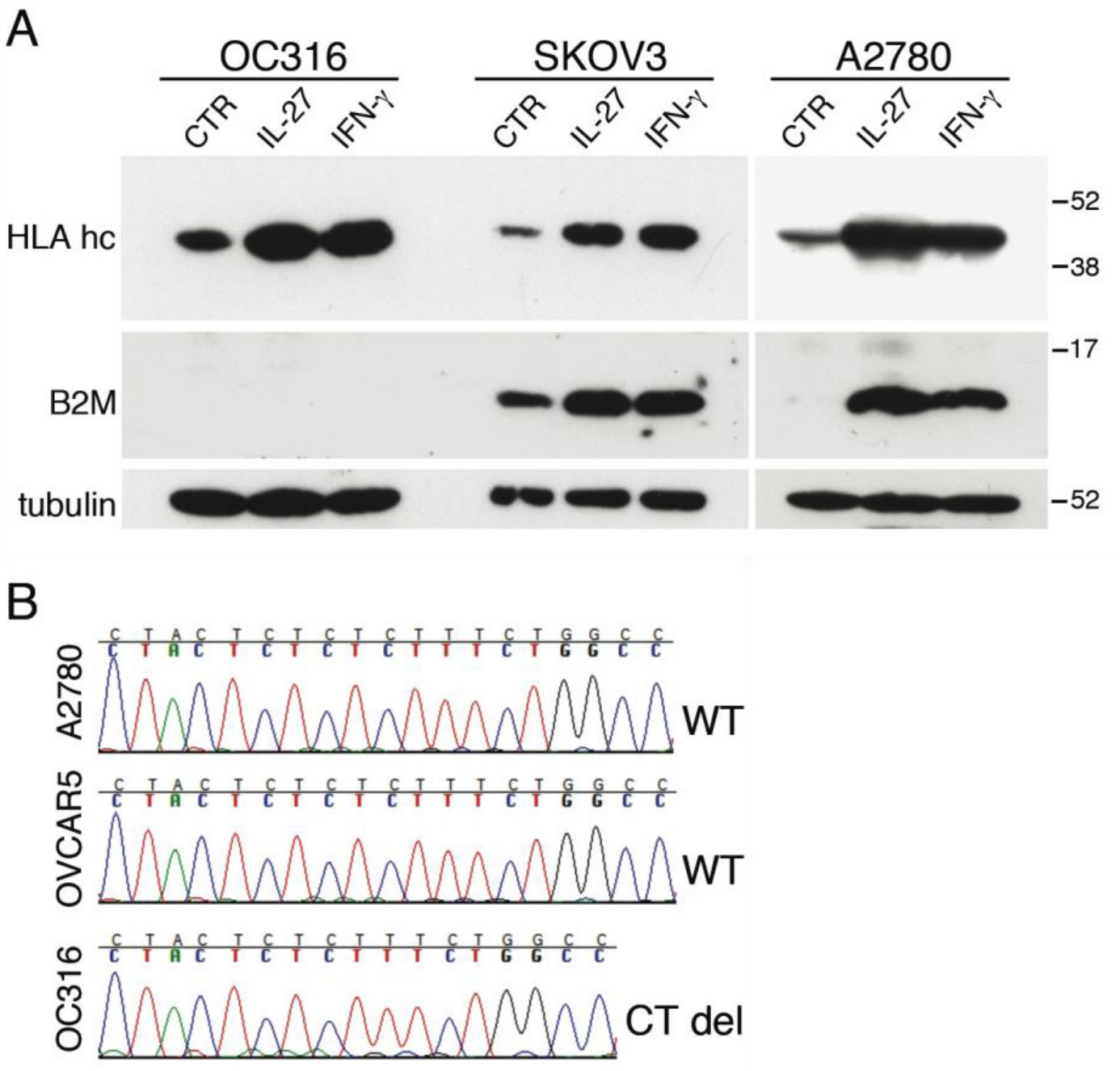
Lack of HLA class I induction in OC316 cells is due to a deletion in the *B2M* gene **A.** Western blot analysis of HLA Class I heavy chains (HLA hc) and β2-microglobulin (B2M) expression in three ovarian cancer cell lines stimulated with the indicated cytokines or medium only (CTR) for 48 hours. α-tubulin is used as loading control. **B.** Sanger sequencing of exons 1 and 2 of the *B2M* gene reveals a bi-allelic CT-deletion in codon 13-15 of exon 1 (CT del) in OC316 cells.

The IL-27-mediated up-regulation of the APM components prompted us to study the effect of IL-27 on the surface expression of HLA class I molecules on a broader panel of cancer cells. This aspect seemed relevant as surface expression of HLA class I on cancer cells is crucial for CTL-mediated anti-tumor immunity and its up-regulation could be involved in the anti-tumor effects of IL-27. Indeed, IL-27 significantly enhanced total HLA class I molecules detected by W6/32 mAb on 5 out of 6 ovarian cancer cell lines (Figure 5). This panel also included a recently derived short-term culture, obtained from one patient ascites (IST-A161). Induction has been particularly evident in the A2780 cell line showing a low baseline HLA class I expression, which was clearly enhanced by IL-27 or IFN-γ (Figure 5). However, the ovarian cancer cell line OC316 showed an HLA class I null phenotype, which could not be modified by cytokine treatment. The proteomic analysis performed on OC316 cells showed that both IFN-γ and IL-27 up-regulated several components of the HLA class I APM but not B2M. B2M is essential for the formation of a stable membrane HLA class I complex, and genetic defects of *B2M* are a common cause of an HLA-null phenotype and immune-escape in different tumors [33, 34]. As no data on the B2M defect were reported for this cell line, we validated proteomic data on OC316 by Western blot analysis. As shown in Figure 6A, OC316 showed no B2M protein expression before or after stimulation with IFN-γ or IL-27, while heavy chains were present and induced by both treatments. B2M was present and up-regulated by cytokine treatment in SKOV3 and A2780 cell lines, regarded as controls. Further genetic analyses showed that OC316 cells have bi-allelic CT deletions in the CT-repetitive region of Exon-1 (codons 13CTCTCTCTT15) (Figure 6B), while exon-2 sequences were wild-type (not shown).

**Figure 7:**
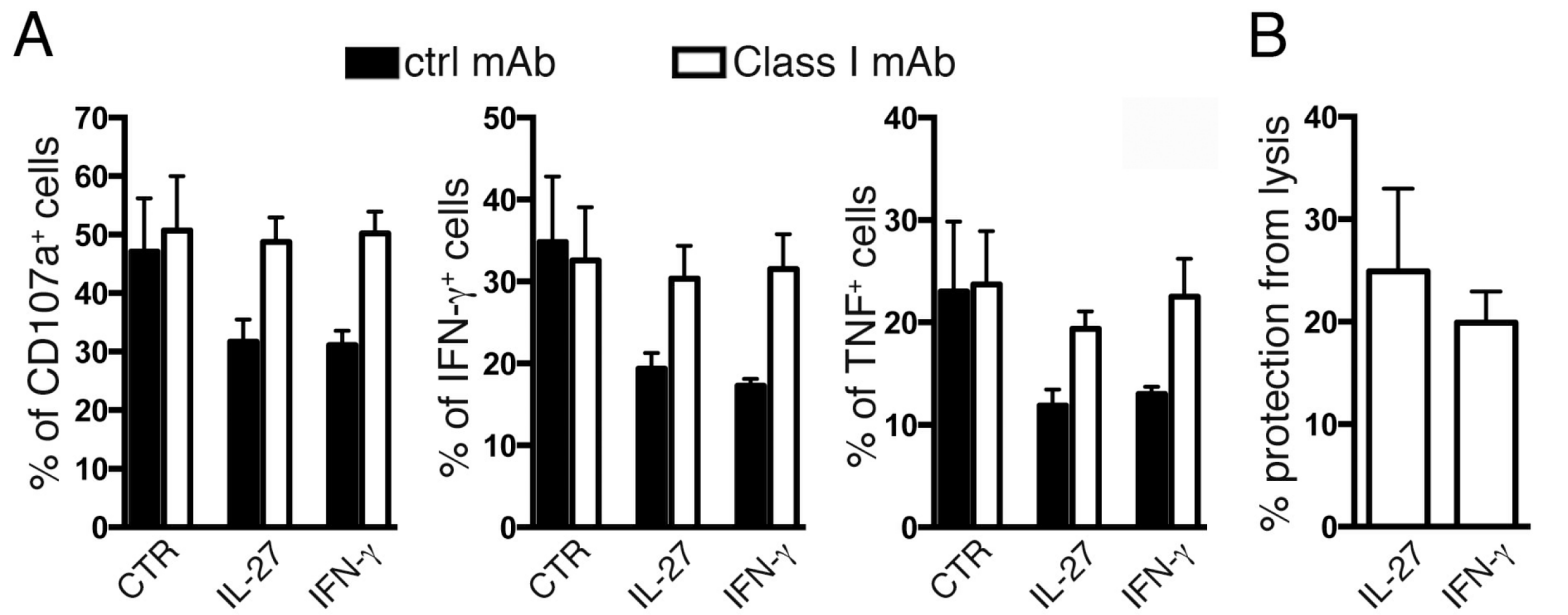
IL-27-mediated up-regulation of HLA class I inhibits NK cell function **A.** Flow cytometric analysis of CD107a expression and IFN-γ and TNF production by long-term IL-2-activated NK cells following 4h co-culture with IL-27- or IFN-γ-treated A2780 tumor cells. The E:T ratio is 1:1. Data are expressed as Mean±SEM. The experiments were performed in the presence (white) or in the absence (black histograms) of a monoclonal antibody specific for HLA class I molecules. **B.** Cytolytic activity (Cr^51^-release assay) of IL-2-activated NK cells against unstimulated or IL-27- or IFN-γ-treated A2780 tumor target cells. Data are expressed as % of protection from NK cell lysis ± SEM of IL-27- or IFN-γ-treated A2780 cells as compared to untreated control. The E:T ratio is 1.2:1. All experiments were performed using polyclonal NK cell populations derived from three different donors.

To verify whether IL-27 up-regulation of surface HLA class I may result in biological effects, we tested whether it may affect NK cell recognition as suggested by pathway analysis. It is well known that HLA class I alleles inhibit NK cell activation through the engagement of their killer immunoglobulin-like receptors (KIRs) [35]. As shown in Figure 7, IL-27 or IFN-γ pre-treatment of A2780 cells resulted in inhibition of NK cell degranulation (CD107a) and cytokine (IFN-γ or TNF) production, as compared to untreated A2780 cells (Figure 7A). Also, NK cytolytic activity was reduced by 24±8 or 20±3% following IL-27 or IFN-γ treatment, respectively (Figure 7B). Masking of HLA class I through an IgM anti-HLA class I antibody completely reverted these effects (Figure 7A).

**Figure 8:**
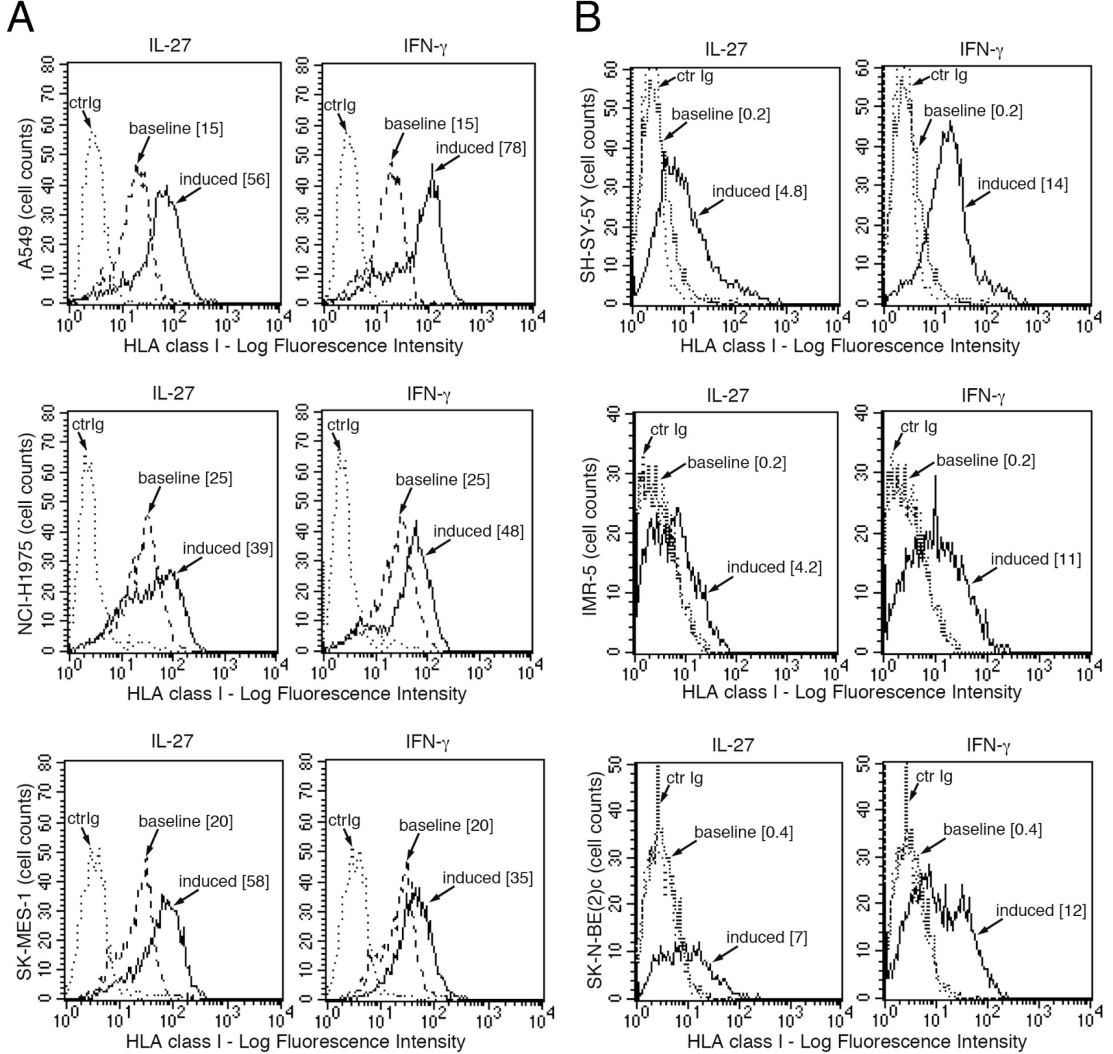
IL-27 increases HLA class I membrane expression in human NSCLC and NB cells *in vitro*. FACS analysis of surface HLA class I expression in three NSCLC **A.** and three neuroblastoma **B.** cell lines, cultured in the presence of medium (baseline), IL-27 or IFN-γ (induced). Isotype-matched unrelated Ig staining control is indicated (ctrIg). Numbers in brackets are Median Fluorescence Intensity (MFI) values calculated as median HLA class I (W6/32 mAb) minus median Ig control.

We then asked whether IL-27 could up-regulate HLA class I expression also on other human cancer cells including three non-small cell lung cancer (NSCLC) and three human neuroblastoma (NB) cell lines. NB cells seemed of particular interest as they frequently display an HLA class I^low^ phenotype due to low expression of different APM components [36]. As shown in Figure 8, IL-27 up-regulated HLA class I expression in the lung cancer cell lines analyzed and induced *de novo* expression in NB cells such as the SH-SY-5Y cell line.

### IL-27-induced protein up-regulation is paralleled by mRNA accumulation, which is mediated by STAT signaling

**Figure 9:**
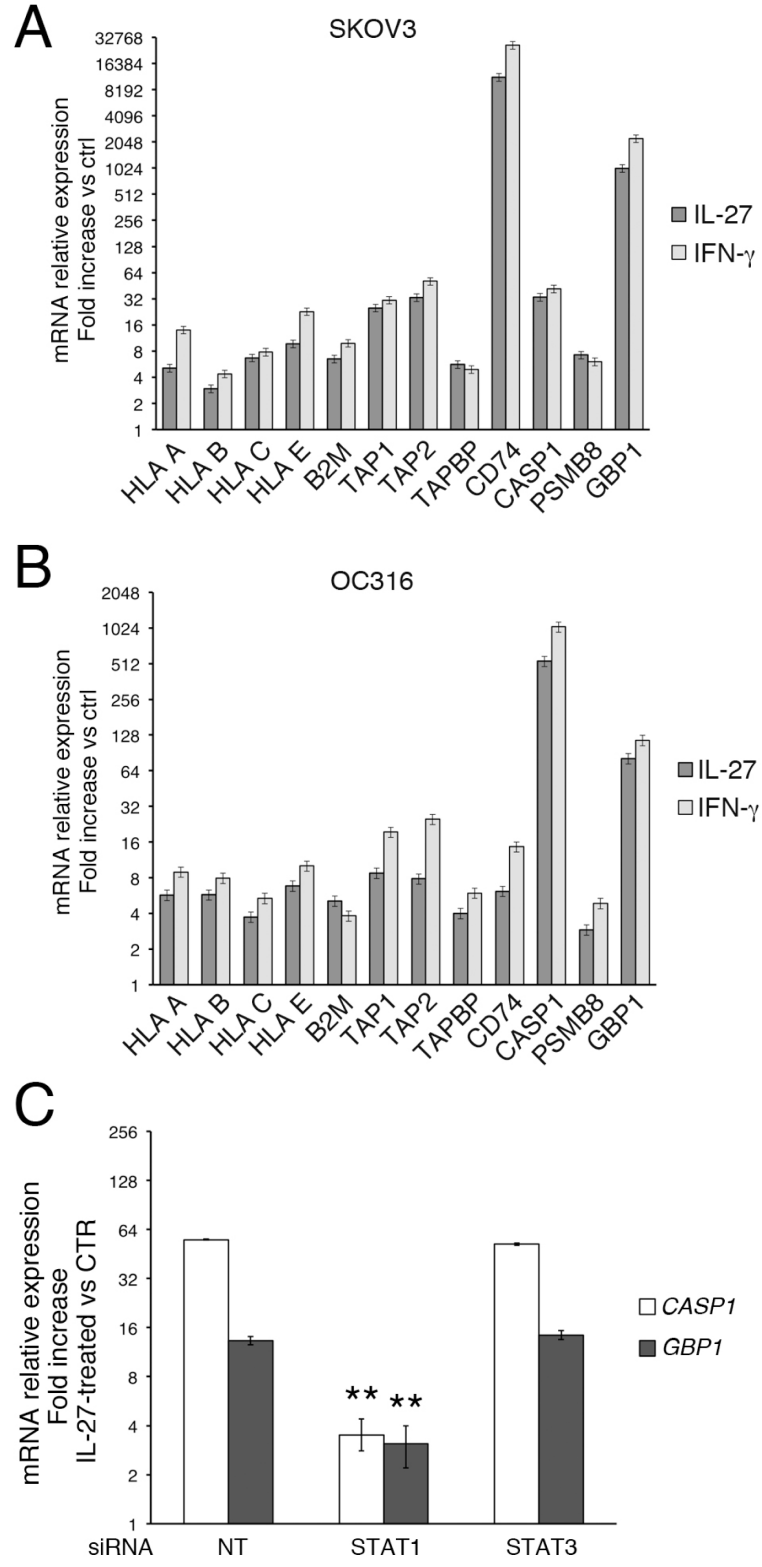
IL-27 increases mRNA expression of representative genes through STAT1 signaling **A.** and **B.** QRT-PCR analysis of mRNA expression in IL-27- and IFN-γ-stimulated cells relative to untreated SKOV3 **A.** and OC316 **B.** cells (ctrl). Data are expressed as fold change relative to control, normalized to *POLR2A* housekeeping gene. Error bars represent SD. **C.** Silencing of STAT1 but not STAT3 with siRNA inhibits IL-27-driven *CASP1* or *GBP1* expression in OC316 cells. *CASP1* and *GBP1* mRNA expression relative to housekeeping gene *POLR2A* mRNA is shown in IL-27-treated OC316 cells transfected with non-targeting (NT), STAT1 or STAT3 siRNA. Data are expressed as ΔΔCT-fold change. Error bars represent SD. ** *P* < 0.0001 *vs* NT sample. A representative experiment out of three with consistent findings is shown.

IL-27 regulates protein expression through STAT1 and STAT3 signaling [2], which are well-known activators of gene transcription. In particular, we previously showed that STAT1 and STAT3 signaling was required for expression of IDO and PD-L1 in ovarian cancer cells [23]. As shown in Figure 9A and 9B, the induction of representative APM class I proteins (HLA-A, -B, -C and -E, B2M, TAP1, TAP2, and TAPBP) and CD74, CASP1, PSMB8 and GBP1 by IL-27 was paralleled by mRNA accumulation, as detected by QRT-PCR analysis. To verify the role of STAT signaling in mRNA accumulation, we also tested IL-27-mediated gene expression in OC316 cells transfected with siRNA targeting STAT1 or STAT3 or non-targeting control siRNA (Figure 9C). These specific siRNAs were effective in reducing total STAT1 or STAT3 protein levels, by 72.5±27.1% or 71.7±12.5%, respectively in four different experiments, as detected by densitometric analyses of Western blots [23]. Our present data show that silencing of STAT1 significantly reduced the accumulation of CASP1 and GBP1 mRNA, two of the most up-regulated mRNAs, in IL-27 treated cells, while STAT3 silencing was ineffective (Figure 9C). Therefore, STAT1 is the principal mediator of IL-27-triggered mRNA expression, at least for genes that show common up-regulation by IFN-γ and IL-27.

## DISCUSSION

The present data indicate that IL-27 profoundly modifies the protein expression profile of human cancer cells and modulates a broad set of proteins in a similar fashion as IFN-γ. Indeed, out of 990 proteins modulated by IL-27, 82% were concordantly modulated by IFN-γ, whereas only about 18% of modulated proteins were differently regulated. Moreover, network analysis showed that IL-27 modulates the expression of proteins involved in IFN signaling and response to IFNs, among which STAT1, IRF-1 and −9, IFIT1, 2 and 3, GBP-1, −2, −4 and −5. STAT1 signaling by IL-27 is well known [2], has been reported in different cellular models and may represent the molecular basis for the broad functional overlap of IFN-γ and IL-27 demonstrated in the present report. As a matter of fact, inhibition of STAT1 expression by specific siRNA diminished the IL-27-triggered expression of GBP1 and CASP1 mRNA, selected as genes similarly regulated by the two cytokines. Importantly, our present report highlights the broad link between IL-27 biological activity and the GBP and IFIT intracellular proteins, which are known mediators of several IFN-γ activities [27-32]. In particular, GBP-1 is one of the most IL-27 up-regulated proteins and is also an important mediator of the IFN-γ-mediated inhibition of endothelial and epithelial tumor cell proliferation, migration, and invasion, which results in anti-angiogenic and anti-tumor effects [29-32]. Parts of these effects involve the GTPase activity of GBP-1, which mediates integrin α4 induction, MMP-1 inhibition, GBP-1 binding to actin and cytoskeleton remodeling [37]. Also, murine mammary carcinoma cells transduced with *GBP1* tet-inducible gene, showed reduced growth into syngeneic mice upon doxycycline treatment, due to reduced angiogenesis and tumor cell proliferation [32]. Our present results suggest that GBP-1 may contribute to IL-27-mediated anti-angiogenic and anti-tumorigenic activities found by others *in vivo* [16-21]. Indeed, IL-27 reduced cell proliferation, as detected by MTT, increased the apoptotic cell rate, and inhibited cell migration of ovarian cancer cell lines *in vitro* (Supplemental Figure S5). However, the pro-apoptotic and anti-proliferative effects of IL-27 were less potent than those of IFN-γ. One may speculate that this difference is related to proteins that are differentially regulated by the two cytokines. Indeed, IL-27 induces activation of STAT3 tyrosine phosphorylation, which may result in pro-survival effects and mitigate STAT1-mediated pro-apoptotic effects.

Also, network analysis indicated that IL-27 may be involved in the negative regulation of viral replication and viral protein expression, through the induction of several IFN-inducible anti-viral proteins (e.g. IFIT 1, 2 and 3, OAS-1 and 3, ISG 15 and 20 and MX1). In agreement with these findings, two previous reports showed that IL-27 displays IFN-γ-like antiviral functions in human hepatocytes [38] and monocyte-derived macrophages [39], which may explain its ability to inhibit HIV-1 replication [40].

Another effect of IL-27 that may contribute to its anti-viral and anti-tumor activities is related to the induction of HLA class I expression. Indeed, network analysis of IL-27-modulated proteins clearly pointed out to a substantial effect on the proteasome and on antigen presentation, peptide folding, assembly and peptide loading of HLA class I. In this context, IL-27 up-regulated the expression of HLA heavy chains of different alleles, B2M, the peptide transporters TAP1 and 2 and the TAP binding protein. It is well known that HLA class I deficiency is a common cause of tumor escape from T cell immunity and may relate to dis-regulation of APM component expression or genetic defects [41]. A down-regulation of HLA class I expression, not related to genetic defects, is also defined as a “soft lesion”, as it can usually be corrected by IFN-γ, which restores CTL recognition in HLA-defective cells [41]. Our present work shows that also IL-27 may correct this defect in cancer cells showing low-HLA class I expression, such as the A2780 ovarian cancer cell line or human neuroblastoma cells. In addition, IL-27 can also up-regulate surface HLA class I levels in different types of human cancer cells already displaying these molecules, including ovarian cancers and NSCLC. This biological activity could contribute to the anti-tumor effects of IL-27, which is mainly related to the induction of Th1 and CTL responses, in pre-clinical models [9, 10, 42]. Other reports showed that IL-27 can modulate MHC class I expression in different cellular contexts. An early report showed that murine neuroblastoma cells engineered to express IL-27 showed delayed tumor formation in syngeneic mice and showed increased MHC class I in the tumor environment due to indirect, IFN-γ-mediated, or possibly direct effects of IL-27 [42]. Also, murine B16F10 melanoma cells transfected with WSX-1 receptor chain up-regulate MHC class I expression [43]. In addition, also human keratinocytes [44], astrocytes [45], and hepatocytes or hepatoma cells [38] or THP-1 monocytic cells [46] display increased HLA class I expression in response to IL-27. On the contrary, IL-27 has been reported to impair HLA class I antigen presentation in human immature DCs [47].

In contrast, complete HLA class I deficiency related to genetic defects has been defined as a “hard lesion”, which cannot be recovered by cytokine treatment and precludes the use of T cell-based immunotherapy strategies [41]. Bi-allelic defects in the *B2M* gene result in total HLA class I loss, as B2M protein is required for the stable membrane expression of HLA heavy chain plus peptide. The CT-deletion in codon 13-15 of exon-1 is a common alteration of this gene in different tumors [33]. Here we show that one out of 6 ovarian cancer cell lines had an IFN-γ- and IL-27-resistant HLA-null phenotype, due to the same defect of the *B2M* gene. Importantly, defective expression of HLA class I components is an independent prognostic marker in ovarian cancers [48, 49]. However, data from the International Cancer Genome Consortium, indicate a low incidence (2.15%) of *B2M* mutations in ovarian cancer tissues, while the frequency was much higher in germinal center B cell lymphomas (19%) and melanoma (11.43% and 13.6%, in two different projects) (https://dcc.icgc.org/genes/ENSG00000166710). Therefore, it is conceivable that *B2M* mutations are a rare cause of HLA deficiency in ovarian cancer and in this respect the B2M-defective OC316 cell line may represent an infrequent genotype of this tumor. Indeed, another study reported that none out of 21 ovarian cancers tested showed defects of B2M, while heavy chain haplotype loss has been reported as a cause of tumor escape in metastases from this tumor [50]. Of note, the W6/32 mAb used in this study recognizes a monomorphic determinant of HLA class I heavy chains bound to B2M and would not allow to define partial HLA class I losses, including haplotype loss. Nonetheless, at least four of the cell lines tested (OVCAR5, SKOV3, A2780 and CAOV3) have no haplotype loss (Supplementary Table S2) [51]. Nowadays, this is a critical issue, as HLA defects may preclude the efficacy of anti-PD-L1 or -PD-1 blocking agents, which are currently undergoing clinical testing in different tumors, including ovarian cancer [52, 53].

Opposite to the induction of protective T cell anti-tumor immunity, the IL-27-mediated induction of HLA class I molecules may result in tumor cell protection from NK cell-mediated lysis as pointed out by network analysis. Indeed, IL-27 pre-treatment of HLA class I^low^ tumor cells inhibited the activation of allogeneic NK cells, as detected by functional assays. Although inhibition of NK cell-mediated killing may not be crucial to the outcome of most solid tumors, this effect may impact on the recognition of hematologic neoplasia, which are more sensitive to NK cell activity [35].

In conclusion, proteomic data indicate that IL-27 shows broadly overlapping functions with IFN-γ, in human cancer cells, most likely in relationship to the common usage of the STAT1 pathway. The effects of this pathway seem to prevail on other signaling pathways as most protein targets show a concordant modulation by the two cytokines, in both ovarian cancer cell lines tested. However, unique activities of IL-27 are suggested by the modulation of a minor, specific set of proteins, possibly due to the specific usage of the STAT3 pathway by IL-27. Noteworthy, the set of proteins specifically modulated by IL-27 differed between SKOV3 and OC316 cells, suggesting that the IL-27-specific effects may diverge according to the cell type. For example, a Gene ontology analysis of IL-27-specific down-regulated proteins in SKOV3 cells, showed a functional relationship to organelle organization.

IL-27 may thus represent a potential tool for immunotherapy not only because it induces Th1/CTL responses *in vivo* [9, 10] but also for its ability to up-regulate HLA class I expression on tumor cells and their recognition by CTLs. However, previous findings suggest a dual role of IL-27 in the anti-tumor immune response [7], which should be taken into account in the design of IL-27-based immunotherapies.

## MATERIALS AND METHODS

### Ethics statement

Investigation has been conducted in accordance with the ethical standards and according to the Declaration of Helsinki and according to national and international guidelines and has been approved by the authors' Institutional Review Board.

### Cells and treatments

Human ovarian cancer cell lines were A2780 and OC316 (ICLC), SKOV3 and CAOV3 (ATCC), OVCAR4 and OVCAR5 (INT Milan). Human NB cell lines were SH-SY-5Y, IMR-5 and SK-N-BE(2)c (ICLC). The lung adenocarcinoma cells A549 and SK-MES1 were from ICLC and NCI-H1975 from ATCC.

Cells were grown, according to suppliers' instructions, either in RPMI 1640, or DMEM or HAM's F-12 supplemented with L-glutamine, 10% FCS and antibiotics (Lonza). A sample of each cell line was recently genotyped using the Cell IDTM System (Promega, G9500) and the GeneMapper^®^ software, version 4.0.

Cells were seeded in 6-well plates at 150×10^3^ cells/well or in 24-well plates at 50×10^3^ cells/well. The day after, the culture medium was replaced with fresh medium with or without recombinant human IFN-γ (1,000 IU/ml, PeproTech, 300-02) or IL-27 (100 ng/ml, R&D System, 2526-IL-010). The culture was then carried on for 48 hours and cells, detached by 2 mM EDTA in PBS, were washed and analyzed by flow cytometry.

IST-A161 cells were derived from a serous ovarian cancer patient by culturing cells from ascites in RPMI 1640 medium supplemented with 4% human AB serum up to passage 10 when they were moved to 10% FCS-containing culture medium. These cells expressed EGFR and Folate Receptor at their surface, while did not express the fibroblast marker CD90 (not shown). Ascites fluids were collected during surgical procedures from patients who gave written informed consent.

Lymphocytes were isolated from peripheral blood of healthy donors using Ficoll-Hypaque density gradient (Lympholyte-H, Cederlane Laboratories Limited). NK cells were enriched using Rosettesep (StemCell Technologies Inc.) and assessed for purity by cytofluorimetric analysis (purity > 97%). To obtain polyclonal activated NK cells, we cultured NK cells on allogeneic irradiated feeder cells in the presence of IL-2 100 U/ml (Proleukin, Chiron) and PHA 1.5 ng/ml (Gibco Life technologies).

### Sample preparation for proteomic analysis

Cell treatments for proteomic analysis were carried out in culture medium with 0.1% FCS. The cells were lysed, solubilized, denatured and reduced using a solution of 4% SDS with 100 mM Tris/HCl pH 7.6, 0.1M DTT. Samples were then processed according to the FASP Protein Digestion Kit instructions (ExpedeonInc, Cat.No. 44250). Briefly, the samples were mixed with 0.2 ml of 8 M urea in 0.1 M Tris/HCl pH 8.5 (UA solution), loaded into the 30 kDa filtration devices, alkylated in 0.1 ml of 50 mM iodoacetamide in UA solution for 20 min in darkness at room temperature (RT).

Sequencing grade trypsin was then added to the samples at a ratio of 1:50 (μg trypsin : μg protein) and incubated overnight at 37°C. After digestion peptides were collected with two washes with 50 mM ammonium bicarbonate. Each digested sample was desalted on StageTips and analyzed by liquid chromatography-tandem mass spectrometry (LC-MS/MS).

### NanoLC setup

The sample was loaded from the sample loop directly into the separation column and the peptides eluted with increasing organic solvent at a flow rate of 250 nl/min. The peptide separations were carried out at 55 °C using a 75-μm ID × 50 cm, 2 μm, 100 Å, C18 column mounted in the thermostated column compartment with a non-linear gradient of 5-65% solution B (80% CAN and 20% H2O, 5% DMSO, 0.1% FA) in 240 min.

### Mass spectrometer setup

The mass spectrometer LTQ-Orbitrap Velos Pro was operated in positive ionization mode. Single MS survey scans were performed in the Orbitrap, recording a mass window between 350 and 1650 m/z using a maximal ion injection time of 250 ms. The resolution was set to 60,000, and the automatic gain control was set to 1,000,000 ions. The lock mass option was enabled allowing the internal recalibration of spectra recorded in the Orbitrap by polydimethylcyclosiloxane background ions (protonated DMSO cluster; m/z 401.922718). The experiments were done in data-dependent acquisition mode with alternating MS and MS/MS experiments. The minimum MS signal for triggering MS/MS was set to 3,000 ions, with the most prominent ion signal selected for MS/MS using an isolation window of 2 Da. The m/z values of signals already selected for MS/MS were put on an exclusion list for 180 s using an exclusion window size of ±5 p.p.m. In all cases, one micro-scan was recorded. CID was done with a target value of 5,000 ions in the linear ion trap, a maximal ion injection time of 150 ms, normalized collision energy of 35%, a Q-value of 0.25 and an activation time of 10 ms. A maximum of 20 MS/MS experiments were triggered per MS scan [54]. The mass spectrometry proteomics data have been deposited to the ProteomeXchange Consortium *via* the PRIDE [55] partner repository with the dataset identifier PXD004419.

### Data analysis

The raw data were processed with MaxQuant software (version 1.5.2.8, http://coxdocs.org/doku.php?id = maxquant:start). It was requested a false discovery rate (FDR) of 0.01 for the identification of proteins, peptides and PSM (peptide-spectrum match) and a minimum length of 6 amino acids for peptide identification. The database used by the software is human (UniProt Release 2014_09) and the digestion enzyme is trypsin [56]. Cysteine carbamidomethylation was selected as fixed modification, whereas acetylation protein N-terminal methionine oxidation and deamidation (N, Q) have been selected as variable modifications. The initial mass deviation for the precursor ion has a tolerance of 7 ppm, while the maximum mass deviation for MS2 events was 0.5 Da. The quantification in MaxQuant was performed using the quantification algorithm MaxLFQ [57] with the option ‘Match between runs’ set to the default parameters.

### Bioinformatics analysis of data

Label-free experiments were analyzed with the Perseus software (http://coxdocs.org/doku.php?id = perseus:start). Protein groups were filtered to require three valid values in at least one experimental group. The label-free intensities were expressed as base log2, and empty values were imputed with random numbers from a normal distribution for each column, to best simulate low abundance values close to noise level. To visualize the profile of the experiment, a modified ANOVA-test was performed with an FDR of 0.05. Hierarchical clustering of resulting proteins was performed on log2 intensities after z-score normalization of the data for each cell line, using Euclidean distances.

To obtain significantly modulated proteins of each comparison (INF-γ *vs* control, IL-27 *vs* control, INF-γ *vs* IL-27) a *t*-test with permutation-based FDR statistics was applied. 250 permutations were performed, with an s0 of 0.5 and required an FDR of 0.05.

To gain information on the main biological processes regulated by IL-27 we performed a comparison of protein data obtained from different treatments (IL-27 and INF-γ). An enrichment analysis based on GO Biological Process and Immunosystem was performed using Cytoscape platform and ClueGO app with these settings: GO Tree Interval: 5-12 to have a detailed network; GO Term Selection: 2 min genes and at least 10% of coverage for each term; evidence code: all without IEA; significance of each term calculated with two-sided hypergeometric with Bonferroni step-down P value correction. A functional network was built from data obtained from this enrichment: big nodes represent GO terms with a *P* < 0.05 while small nodes are query proteins associated with the terms obtained from the analysis.

### Evaluation of cell proliferation and apoptosis

Cell viability and proliferation after treatment with IL-27 or IFN-γ was evaluated by a microculture tetrazolium reduction assay using MTT [3-(4,5-dimethylthiazol-2-yl) 2,5-diphenyltetrazolium bromide; Sigma M2128] following standard protocols. One to 2×10^3^ cells per well in 100 μl of culture medium was seeded in 96-wells flat-bottom plates. Each experimental condition was replicated in six wells. The day after, 100 μl of medium containing the cytokines were added and culture was carried on for 4 to 6 days, according to the different cell types. Twenty μl of MTT stock solution (2 mg/ml in PBS) was added for the last 4 hours of culture and precipitated formazan was then dissolved in 100 μl of DMSO. Data are expressed as percentage of control samples.

Apoptotic cell death was analyzed by the Annexin V-FITC/PI Apoptosis Detection Kit (eBioscience DX Diagnostics BMS500FI/300CE, Bender MedSystems) and flow cytometry, according to the manufacturer's instructions. Ten to 40×10^3^ cells were seeded in 24-wells plates in 0.5 ml culture medium. The next day, culture medium was replaced with medium containing the different cytokines for additional 3 to 6 days, according to the different cell proliferation rate.

### Immunofluorescence

Indirect immunofluorescence was performed on 50 to 100×10^3^ cells/sample with anti-HLA class I W6/32 mAb (ATCC) and FITC-labeled goat anti-mouse (Jackson Immunoresearch, 115-096-068) according to standard techniques. Immunofluorescence was analyzed in a FACScan (Becton&Dickinson) with the Cell Quest software, by gating on viable cells and acquisition of 10^4^ gated events.

### RT-PCR analysis

Total RNA was isolated by the NucleoSpin RNA kit (Macherey-Nagel, 740955.250) and reverse-transcribed using the SuperScript III Reverse Transcriptase (Invitrogen, 18064-071). The primers used for Quantitative (Q)RT-PCR analysis are listed in the supplementary material (Table S1). Amplification was performed using the iQTM SYBR^®^ Green Supermix system (Bio-Rad Laboratories, 170-8882) in the Mastercycler^®^ ep realplex4 instrument (Eppendorf International). Relative quantification of mRNAs was calculated by the ΔΔCt method.

### Western blot

Cells were lysed with 20 mM Tris-HCl pH 7.4, 1 mM EDTA, 150 mM NaCl, 1% Brij97, 2 mM Na Orthovanadate and protease inhibitors (Roche Diagnostics, Complete Mini 04693124001). Proteins were resolved by 12% SDS-PAGE under reducing conditions and Western blotting according to standard techniques. Monoclonal antibodies anti-HLA class I heavy chain (clone TP25.99) and anti-β2 microglobulin (clone NAMB-1) were kindly provided by S. Ferrone. Immunoreactive proteins were detected by ECL Prime (GE Healthcare, RPN2232) and autoradiography.

### Small Interfering RNA (siRNA)-mediated STAT1 or STAT3 silencing

iBONIsiRNA-Pool targeting human STAT1 or STAT3 mRNA or Non-Targeting control siRNA pool (RIBOXX Life Science) and Lipofectamine 2000 Reagent (Invitrogen, 11668-019) were used to transfect OC316 ovarian cancer cells as described elsewhere [23]. Cell treatments were performed as above described.

### Sanger sequencing

Sanger sequencing of exons 1 and 2 of *B2M* was carried out using the following primers: primer F exon 1: GCCTGAAGTCCTAGAATGAGC; primer R exon 1: CCGAAAGGGGCAAGTAGC; Primer F exon 2: TCACGGTTTATTCTTCAAAATGG; Primer R exon 2: GGGATGGGACTCATTCAGG. Both primer pairs were designed by Primer3. Purified PCR products (ExoSAP-IT GE-Healthcare) were sequenced bi-directionally using the by BigDye Terminator v1.1 kit (Life Technologies) on the ABI-Prism 3130 genetic analyzer (Life Technologies). Outputs were analyzed using the Sequencher 5.1 software.

### Degranulation analysis and cytokine secretion

To study degranulation activity (CD107a expression) and cytokines production (IFN-γ and TNF), polyclonal activated NK cells were cultured with untreated, IL-27- or IFN-γ-stimulated (48h) A2780 cells for 4 hours in the presence or in the absence of anti-HLA class I mAb (A6136, IgM final concentration 10 μg/ml, kindly provided by A. Moretta, Italy). An Effector:Target (E:T) ratio of 1:1 was used. To detect spontaneous degranulation, a control sample without target cells was included. Monensin-containing GolgiStop (BD Biosciences) at final concentration of 2 mM was added and surface/intracellular staining was performed. All samples were analyzed on a Gallios Flow Cytometer (Beckman Coulter). Data analysis was done using FlowJo software (TreeStar Inc.).

### Cytolytic activity

Polyclonal activated NK cells were tested for cytolytic activity in a 4h Cr^51^-release assay against A2780 cell line. In masking experiments, target cells were pre-incubated with anti-HLA class I specific mAb. Different E:T ratio was used.

## SUPPLEMENTARY MATERIALS FIGURES AND TABLES



## References

[1] Pflanz S, Timans JC, Cheung J, Rosales R, Kanzler H, Gilbert, Hibbert L, Churakova T, Travis M, Vaisberg E, Blumenschein WM, Mattson JD, Wagner JL (2002). IL-27, a heterodimeric cytokine composed of EBI3 and p28 protein, induces proliferation of naive CD4+ T cells. Immunity.

[2] Vignali DA, Kuchroo VK (2012). IL-12 family cytokines: immunological playmakers. Nature immunology.

[3] Trinchieri G, Pflanz S, Kastelein RA (2003). The IL-12 family of heterodimeric cytokines: new players in the regulation of T cell responses. Immunity.

[4] Pirhonen J, Siren J, Julkunen I, Matikainen S (2007). IFN-alpha regulates Toll-like receptor-mediated IL-27 gene expression in human macrophages. Journal of leukocyte biology.

[5] Hause L, Al-Salleeh FM, Petro TM (2007). Expression of IL-27 p28 by Theiler's virus-infected macrophages depends on TLR3 and TLR7 activation of JNK-MAP-kinases. Antiviral Research.

[6] Wirtz S, Becker C, Fantini MC, Nieuwenhuis EE, Tubbe I, Galle PR, Schild HJ, Birkenbach M, Blumberg RS, Neurath MF (2005). EBV-induced gene 3 transcription is induced by TLR signaling in primary dendritic cells *via* NF-kappa B activation. Journal of immunology (Baltimore, Md: 1950).

[7] Li MS, Liu Z, Liu JQ, Zhu X, Liu Z, Bai XF (2015). The Yin and Yang aspects of IL-27 in induction of cancer-specific T-cell responses and immunotherapy. Immunotherapy.

[8] Lucas S, Ghilardi N, Li J, de Sauvage FJ (2003). IL-27 regulates IL-12 responsiveness of naive CD4+ T cells through Stat1-dependent and -independent mechanisms.

[9] Morishima N, Owaki T, Asakawa M, Kamiya S, Mizuguchi J, Yoshimoto T (2005). Augmentation of effector CD8+ T cell generation with enhanced granzyme B expression by IL-27. Journal of immunology (Baltimore, Md: 1950).

[10] Liu Z, Liu JQ, Talebian F, Wu LC, Li S, Bai XF (2013). IL-27 enhances the survival of tumor antigen-specific CD8+ T cells and programs them into IL-10-producing, memory precursor-like effector cells. European journal of immunology.

[11] Awasthi A, Carrier Y, Peron JP, Bettelli E, Kamanaka M, Flavell RA, Kuchroo VK, Oukka M, Weiner HL (2007). A dominant function for interleukin 27 in generating interleukin 10-producing anti-inflammatory T cells. Nature immunology.

[12] Batten M, Li J, Yi S, Kljavin NM, Danilenko DM, Lucas S, Lee J, de Sauvage FJ, Ghilardi N (2006). Interleukin 27 limits autoimmune encephalomyelitis by suppressing the development of interleukin 17-producing T cells. Nature immunology.

[13] Hirahara K, Ghoreschi K, Yang XP, Takahashi H, Laurence A, Vahedi G, Sciume G, Hall AO, Dupont CD, Francisco LM, Chen Q, Tanaka M, Kanno Y (2012). Interleukin-27 priming of T cells controls IL-17 production in trans *via* induction of the ligand PD-L1. Immunity.

[14] Apetoh L, Quintana FJ, Pot C, Joller N, Xiao S, Kumar D, Burns EJ, Sherr DH, Weiner HL, Kuchroo VK (2010). The aryl hydrocarbon receptor interacts with c-Maf to promote the differentiation of type 1 regulatory T cells induced by IL-27. Nature immunology.

[15] Sekar D, Hahn C, Brune B, Roberts E, Weigert A (2012). Apoptotic tumor cells induce IL-27 release from human DCs to activate Treg cells that express CD69 and attenuate cytotoxicity. European journal of immunology.

[16] Zorzoli A, Di Carlo E, Cocco C, Ognio E, Ribatti D, Ferretti E, Dufour C, Locatelli F, Montagna D, Airoldi I (2012). Interleukin-27 inhibits the growth of pediatric acute myeloid leukemia in NOD/SCID/Il2rg−/− mice. Clinical cancer research.

[17] Canale S, Cocco C, Frasson C, Seganfreddo E, Di Carlo E, Ognio E, Sorrentino C, Ribatti D, Zorzoli A, Basso G, Dufour C, Airoldi I (2011). Interleukin-27 inhibits pediatric B-acute lymphoblastic leukemia cell spreading in a preclinical model. Leukemia.

[18] Cocco C, Giuliani N, Di Carlo E, Ognio E, Storti P, Abeltino M, Sorrentino C, Ponzoni M, Ribatti D, Airoldi I (2010). Interleukin-27 acts as multifunctional antitumor agent in multiple myeloma. Clinical cancer research.

[19] Di Carlo E, Sorrentino C, Zorzoli A, Di Meo S, Tupone MG, Ognio E, Mincione G, Airoldi I (2014). The antitumor potential of Interleukin-27 in prostate cancer. Oncotarget.

[20] Shimizu M, Shimamura M, Owaki T, Asakawa M, Fujita K, Kudo M, Iwakura Y, Takeda Y, Luster AD, Mizuguchi J, Yoshimoto T (2006). Antiangiogenic and antitumor activities of IL-27. Journal of immunology (Baltimore, Md: 1950).

[21] Airoldi I, Tupone MG, Esposito S, Russo MV, Barbarito G, Cipollone G, Di Carlo E (2015). Interleukin-27 re-educates intratumoral myeloid cells and down-regulates stemness genes in non-small cell lung cancer. Oncotarget.

[22] Carbotti G, Barisione G, Orengo AM, Brizzolara A, Airoldi I, Bagnoli M, Pinciroli P, Mezzanzanica D, Centurioni MG, Fabbi M, Ferrini S (2013). The IL-18 antagonist IL-18-binding protein is produced in the human ovarian cancer microenvironment. Clinical cancer research.

[23] Carbotti G, Barisione G, Airoldi I, Mezzanzanica D, Bagnoli M, Ferrero S, Petretto A, Fabbi M, Ferrini S (2015). IL-27 induces the expression of IDO and PD-L1 in human cancer cells. Oncotarget.

[24] Alama A, Barbieri F, Favre A, Cagnoli M, Noviello E, Pedulla F, Viale M, Foglia G, Ragni N (1996). Establishment and characterization of three new cell lines derived from the ascites of human ovarian carcinomas. Gynecologic oncology.

[25] Chin YE, Kitagawa M, Kuida K, Flavell RA, Fu XY (1997). Activation of the STAT signaling pathway can cause expression of caspase 1 and apoptosis. Molecular and cellular biology.

[26] Ozato K, Tailor P, Kubota T (2007). The interferon regulatory factor family in host defense: mechanism of action. The Journal of biological chemistry.

[27] Diamond MS, Farzan M (2013). The broad-spectrum antiviral functions of IFIT and IFITM proteins. Nature reviews. Immunology.

[28] Meunier E, Broz P (2016). Interferon-inducible GTPases in cell autonomous and innate immunity. Cellular microbiology.

[29] Anderson SL, Carton JM, Lou J, Xing L, Rubin BY (1999). Interferon-induced guanylate binding protein-1 (GBP-1) mediates an antiviral effect against vesicular stomatitis virus and encephalomyocarditis virus. Virology.

[30] Guenzi E, Topolt K, Cornali E, Lubeseder-Martellato C, Jorg A, Matzen K, Zietz C, Kremmer E, Nappi F, Schwemmle M, Hohenadl C, Barillari G, Tschachler E (2001). The helical domain of GBP-1 mediates the inhibition of endothelial cell proliferation by inflammatory cytokines. The EMBO journal.

[31] Weinlander K, Naschberger E, Lehmann MH, Tripal P, Paster W, Stockinger H, Hohenadl C, Sturzl M (2008). Guanylate binding protein-1 inhibits spreading and migration of endothelial cells through induction of integrin alpha4 expression. FASEB journal.

[32] Lipnik K, Naschberger E, Gonin-Laurent N, Kodajova P, Petznek H, Rungaldier S, Astigiano S, Ferrini S, Sturzl M, Hohenadl C (2010). Interferon gamma-induced human guanylate binding protein 1 inhibits mammary tumor growth in mice. Molecular medicine (Cambridge, Mass.).

[33] Bernal M, Ruiz-Cabello F, Concha A, Paschen A, Garrido F (2012). Implication of the beta2-microglobulin gene in the generation of tumor escape phenotypes. Cancer immunology, immunotherapy.

[34] Garrido F, Romero I, Aptsiauri N, Garcia-Lora AM (2016). Generation of MHC class I diversity in primary tumors and selection of the malignant phenotype. International journal of cancer.

[35] Moretta L, Pietra G, Montaldo E, Vacca P, Pende D, Falco M, Del Zotto G, Locatelli F, Moretta A, Mingari MC (2014). Human NK cells: from surface receptors to the therapy of leukemias and solid tumors. Frontiers in immunology.

[36] Corrias MV, Occhino M, Croce M, De Ambrosis A, Pistillo MP, Bocca P, Pistoia V, Ferrini S (2001). Lack of HLA-class I antigens in human neuroblastoma cells: analysis of its relationship to TAP and tapasin expression. Tissue antigens.

[37] Ostler N, Britzen-Laurent N, Liebl A, Naschberger E, Lochnit G, Ostler M, Forster F, Kunzelmann P, Ince S, Supper V, Praefcke GJ, Schubert DW, Stockinger H (2014). Gamma interferon-induced guanylate binding protein 1 is a novel actin cytoskeleton remodeling factor. Molecular and cellular biology.

[38] Bender H, Wiesinger MY, Nordhoff C, Schoenherr C, Haan C, Ludwig S, Weiskirchen R, Kato N, Heinrich PC, Haan S (2009). Interleukin-27 displays interferon-gamma-like functions in human hepatoma cells and hepatocytes. Hepatology (Baltimore, Md.).

[39] Imamichi T, Yang J, Huang DW, Brann TW, Fullmer BA, Adelsberger JW, Lempicki RA, Baseler MW, Lane HC (2008). IL-27, a novel anti-HIV cytokine, activates multiple interferon-inducible genes in macrophages. AIDS (London, England).

[40] Fakruddin JM, Lempicki RA, Gorelick RJ, Yang J, Adelsberger JW, Garcia-Pineres AJ, Pinto LA, Lane HC, Imamichi T (2007). Noninfectious papilloma virus-like particles inhibit HIV-1 replication: implications for immune control of HIV-1 infection by IL-27. Blood.

[41] Garrido F, Cabrera T, Aptsiauri N (2010). “Hard” and “soft” lesions underlying the HLA class I alterations in cancer cells: implications for immunotherapy. International journal of cancer.

[42] Salcedo R, Stauffer JK, Lincoln E, Back TC, Hixon JA, Hahn C, Shafer-Weaver K, Malyguine A, Kastelein R, Wigginton JM (2004). IL-27 mediates complete regression of orthotopic primary and metastatic murine neuroblastoma tumors: role for CD8+ T cells. Journal of immunology (Baltimore, Md.: 1950).

[43] Yoshimoto T, Morishima N, Mizoguchi I, Shimizu M, Nagai H, Oniki S, Oka M, Nishigori C, Mizuguchi J (2008). Antiproliferative activity of IL-27 on melanoma. Journal of immunology (Baltimore, Md.: 1950).

[44] Wittmann M, Zeitvogel J, Wang D, Werfel T (2009). IL-27 is expressed in chronic human eczematous skin lesions and stimulates human keratinocytes. The Journal of allergy and clinical immunology.

[45] Senecal V, Deblois G, Beauseigle D, Schneider R, Brandenburg J, Newcombe J, Moore CS, Prat A, Antel J, Arbour N (2016). Production of IL-27 in multiple sclerosis lesions by astrocytes and myeloid cells: Modulation of local immune responses. Glia.

[46] Feng XM, Liu N, Yang SG, Hu LY, Chen XL, Fang ZH, Ren Q, Lu SH, Liu B, Han ZC (2008). Regulation of the class II and class I MHC pathways in human THP-1 monocytic cells by interleukin-27. Biochemical and biophysical research communications.

[47] Morandi F, Di Carlo E, Ferrone S, Petretto A, Pistoia V, Airoldi I (2014). IL-27 in human secondary lymphoid organs attracts myeloid dendritic cells and impairs HLA class I-restricted antigen presentation. Journal of immunology (Baltimore, Md.: 1950).

[48] Han LY, Fletcher MS, Urbauer DL, Mueller P, Landen CN, Kamat AA, Lin YG, Merritt WM, Spannuth WA, Deavers MT, De Geest K, Gershenson DM, Lutgendorf SK (2008). HLA class I antigen processing machinery component expression and intratumoral T-Cell infiltrate as independent prognostic markers in ovarian carcinoma. Clinical cancer research.

[49] Rolland P, Deen S, Scott I, Durrant L, Spendlove I (2007). Human leukocyte antigen class I antigen expression is an independent prognostic factor in ovarian cancer. Clinical cancer research.

[50] Norell H, Carlsten M, Ohlum T, Malmberg KJ, Masucci G, Schedvins K, Altermann W, Handke D, Atkins D, Seliger B, Kiessling R (2006). Frequent loss of HLA-A2 expression in metastasizing ovarian carcinomas associated with genomic haplotype loss and HLA-A2-restricted HER-2/neu-specific immunity. Cancer research.

[51] Scholtalbers J, Boegel S, Bukur T, Byl M, Goerges S, Sorn P, Loewer M, Sahin U, Castle JC (2015). TCLP: an online cancer cell line catalogue integrating HLA type, predicted neo-epitopes, virus and gene expression. Genome medicine.

[52] Callahan MK, Postow MA, Wolchok JD (2016). Targeting T Cell Co-receptors for Cancer Therapy. Immunity.

[53] Zhu X, Lang J (2016). The significance and therapeutic potential of PD-1 and its ligands in ovarian cancer: A systematic review. Gynecologic oncology.

[54] Kocher T, Pichler P, Swart R, Mechtler K (2012). Analysis of protein mixtures from whole-cell extracts by single-run nanoLC-MS/MS using ultralong gradients. Nature protocols.

[55] Vizcaino JA, Csordas A, del-Toro N, Dianes JA, Griss J, Lavidas I, Mayer G, Perez-Riverol Y, Reisinger F, Ternent T, Xu QW, Wang R, Hermjakob H (2016). 2016 update of the PRIDE database and its related tools. Nucleic acids research.

[56] Eberl HC, Spruijt CG, Kelstrup CD, Vermeulen M, Mann M (2013). A map of general and specialized chromatin readers in mouse tissues generated by label-free interaction proteomics. Molecular cell.

[57] Cox J, Hein MY, Luber CA, Paron I, Nagaraj N, Mann M (2014). Accurate proteome-wide label-free quantification by delayed normalization and maximal peptide ratio extraction, termed MaxLFQ. Molecular & cellular proteomics.

