# Supplementary material for: Proteomic analysis uncovers common effects of IFN-γ and IL-27 on the HLA class I antigen presentation machinery in human cancer cells
Source: Oncotarget. 2016 Sep 24;7(45):72518–36. doi: 10.18632/oncotarget.12235 (PMC5341926; doi:10.18632/oncotarget.12235)
Supplement: Supplementary file 1 [file oncotarget-07-72518-s001.pdf]

Proteomic analysis uncovers common effects of IFN-γ and IL-27 on the HLA class I antigen presentation machinery in human cancer cells

Supplementary Material

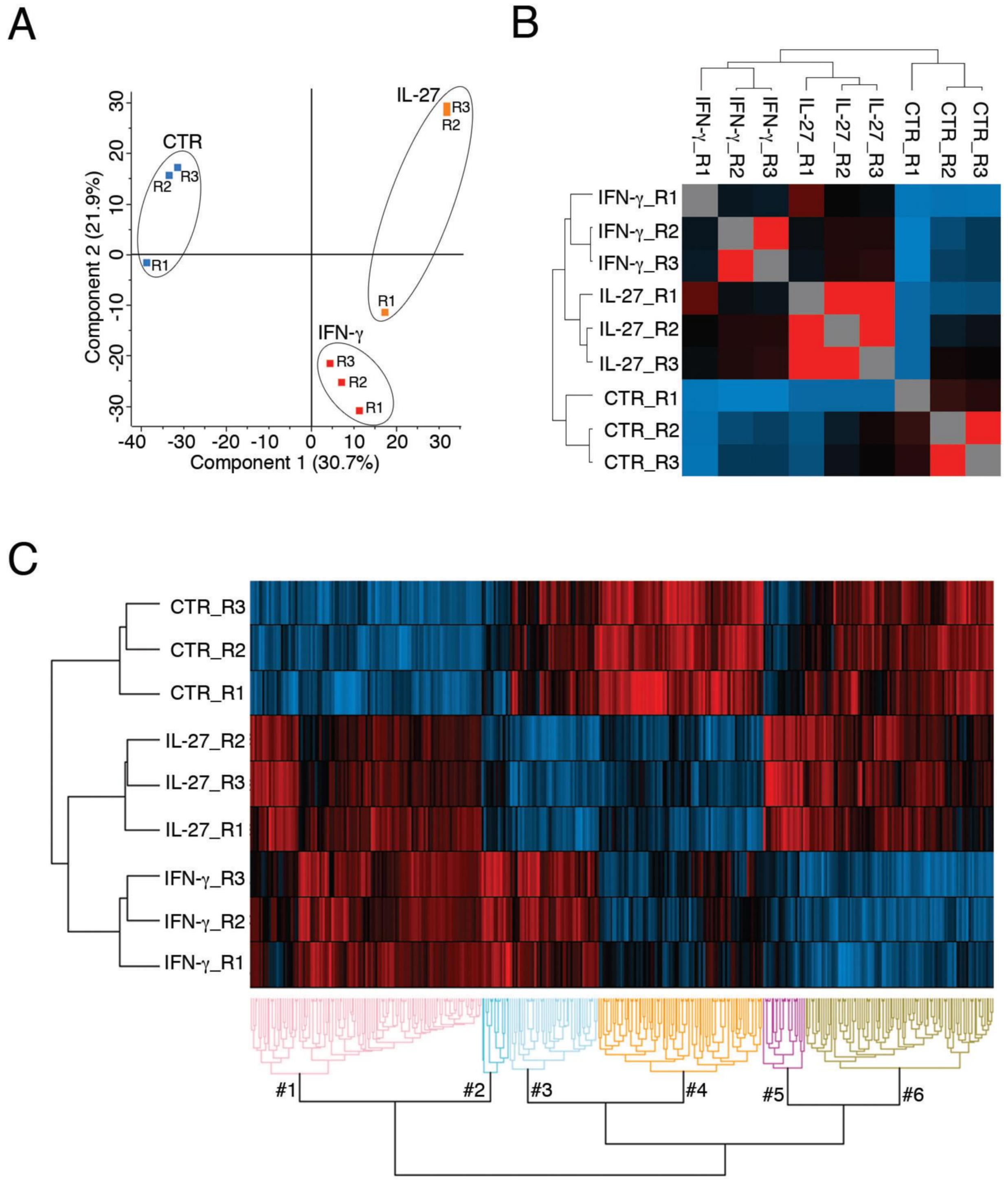

Figure S1: Principal component analysis, Pearson's correlogram and unsupervised hierarchical clustering analysis of untreated, IFN-γ- or IL-27-treated OC316 cells

A: Two-dimensional scatter plot of the principal component analysis of OC316 Untreated (blue),

IFN-γ - (red) and IL-27-treated (orange squares) samples.
B: The Pearson's correlogram depicts the coefficient values in a pseudo-color scale, which extends from 0.1 (light blue) to 0.9 (red). The dendrogram displays the results of an unsupervised hierarchical clustering analysis placing similar Pearson's coefficient values near each other. All the samples cluster according to treatment.
C: Unsupervised hierarchical clustered heatmap of 370 proteins identified by Multiple-samples test ANOVA performed on the OC316 cell line. The amount of each protein in individual samples is represented by the color scheme in which red and blue indicate high and low expression of proteins, respectively. Three independent biological replicates of cells treated with IL-27 or IFN-γ or left untreated (CTR) are shown. Proteins are clustered into 6 groups according to their expression value. Cluster #1 and cluster #4 represent a concordant modulation by IFN-γ and IL-27 treatments. In particular, 115 proteins were up-regulated in cluster #1 and 82 were down-regulated in cluster #4 by both cytokines. A smaller number of proteins (173) was differentially modulated by the two cytokines and clustered into 4 small groups, relative to untreated cells.

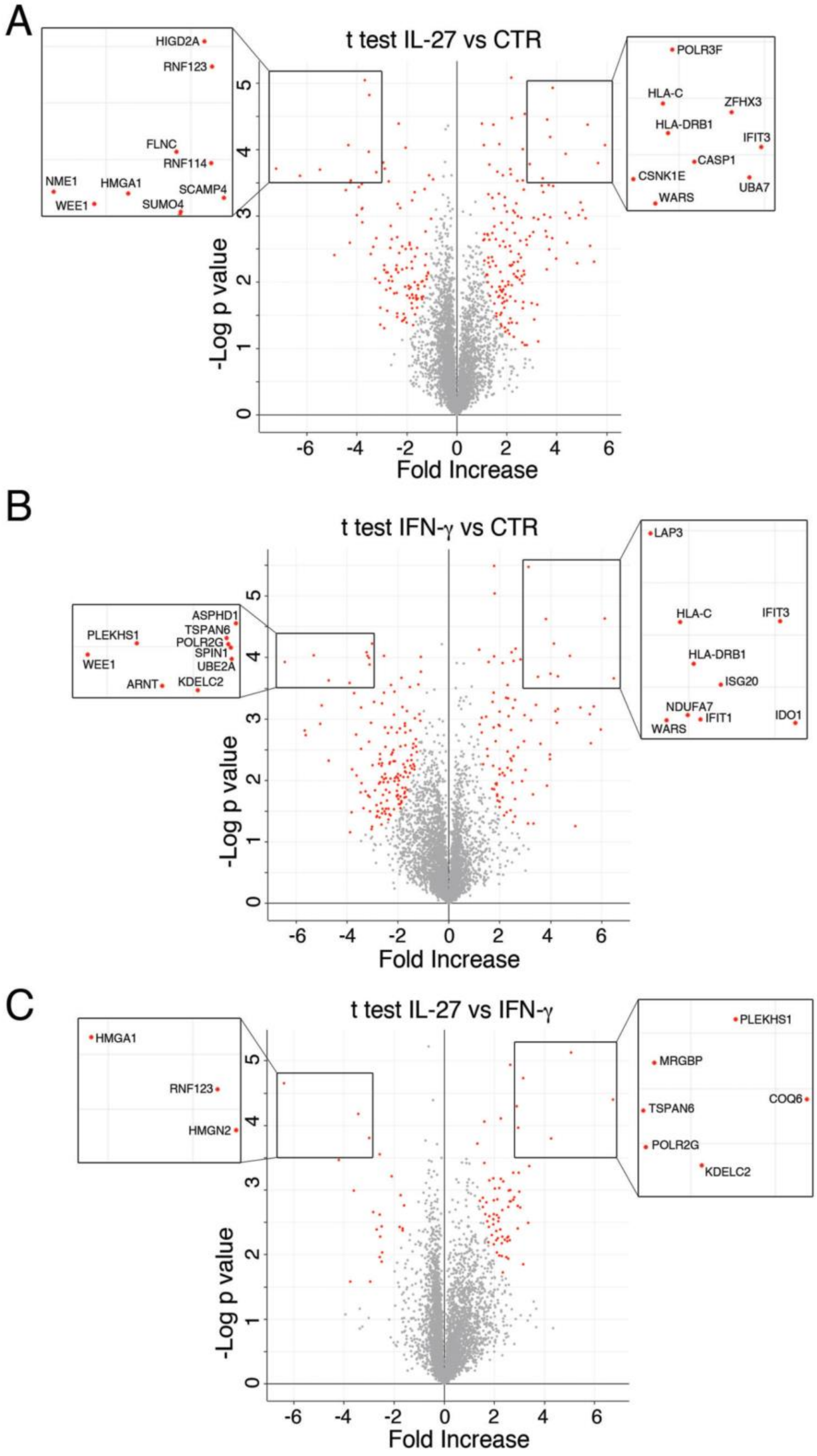

Figure S2: Volcano plot representation of differentially expressed proteins
Plots represent untreated (CTR) and IL-27-treated (A), CTR and INF- γ -treated (B) and IL-27 and IFN- γ -treated (C) OC316 cell samples. Red dots represent proteins that display both large magnitude fold-changes (x-axis, to the right there are proteins up-regulated after treatment) as well as high statistical significance (-log10 of p-value, y-axis). The black-line shows where FDR=0.05 and s0=0.5. Gray dots represent proteins whose fold-change is <2 (log2 = 1) or the p-value >0.05. Proteins labeled with gene name (inset) are the most modulated ones.

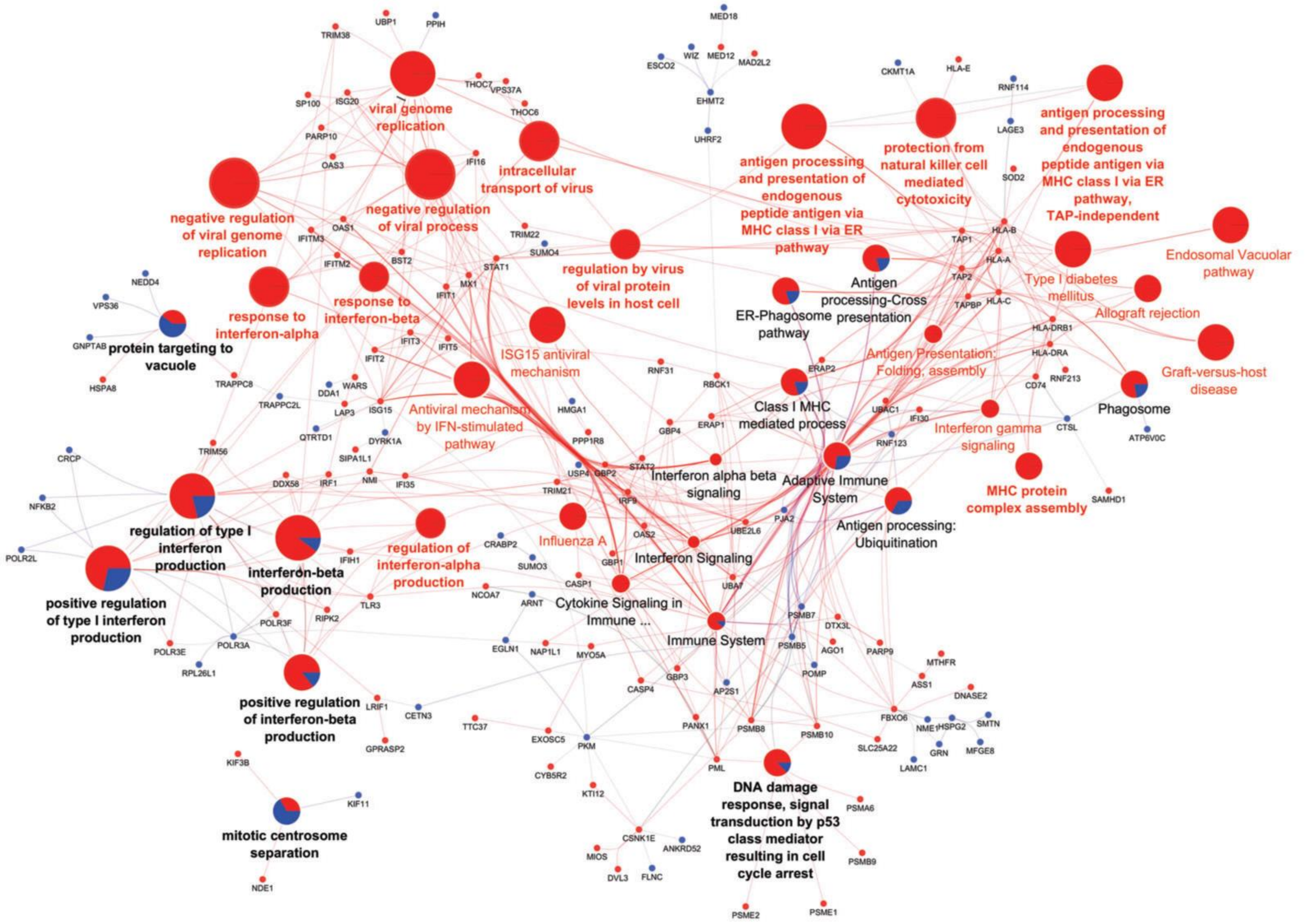

Figure S3: Functional networks of IL-27-modulated proteins in OC316 cells
The network was obtained from proteins showing significant (t-test) variations between IL-27 treatment and control, through the merge between a first network obtained according to ClueGO setup and a second one built by GENEMANIA app. Each big node represents a Gene Ontology Biological Process term or a Reactome pathway, while the small node represents query proteins used for the analysis from which previous terms are obtained. The node size represents the P-value obtained from two-side hypergeometric test corrected by the Bonferroni step-down method. Red and blue indicate high and low expression of proteins. The same colors are used for the node terms in which the pie represents the percentage of protein up or down regulated.

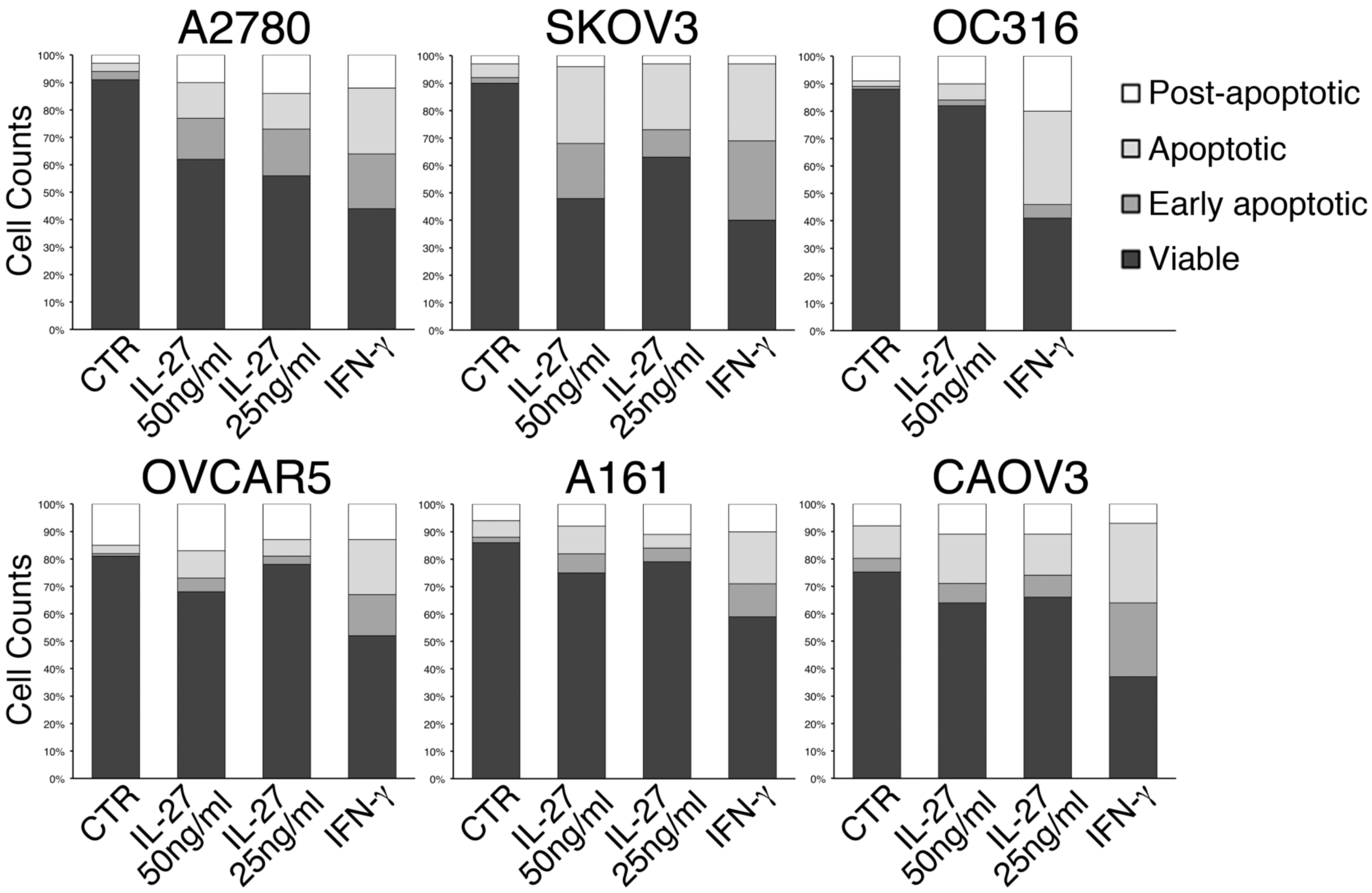

Figure S4: IL-27 induces apoptosis in most ovarian cancer cell lines.
Cells were treated with or without IL-27 at the concentrations indicated for 48-72 hours and then apoptosis was measured by the Annexin V-FITC/PI staining and cytofluorimetric analysis. IFN- γ (1000U/ml) was used as positive control in parallel cultures.

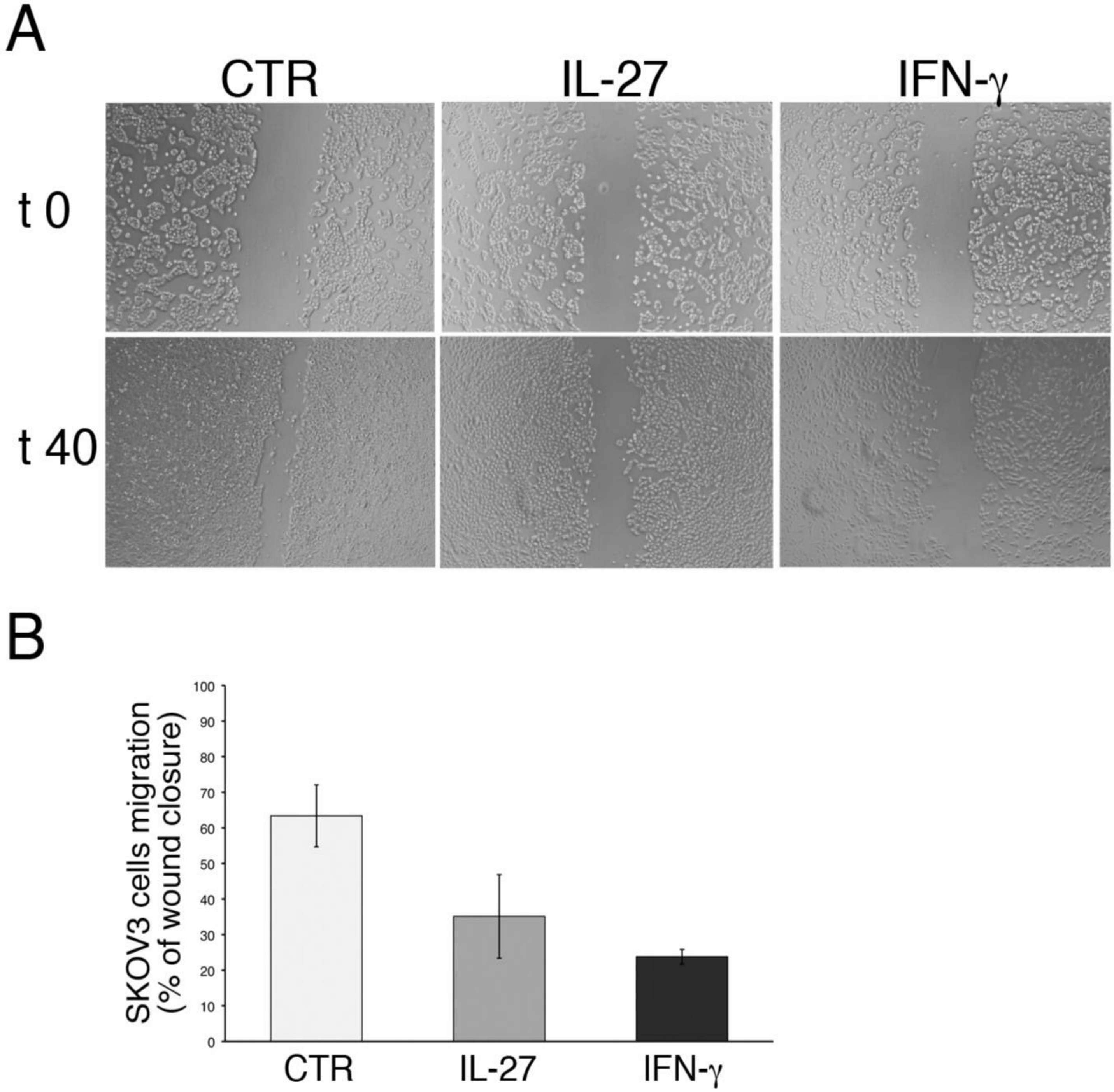

Figure S5: IL-27 inhibits SKOV3 cell migration in a wound-healing assay.
IL-27 (100 ng/ml) or IFN-γ (1000U/ml) reduce cell migration ($P<0.05$) with respect to the untreated control in a wound-healing assay on SKOV3 cells. Data are expressed as mean±SD percent of a representative experiment performed in triplicate. Consistent data were obtained in one additional experiment.

TABLE S1. QRT-PCR primers

Gene	Forward primer	Reverse primer
GAPDH	GAAGGTGAAGGTCGGAGT	CATGGGTGGAATCATATTGGAA
POLR2A	GACAATGCAGAGAAGCTGG	GCAGGAAGACATCATCATCC
HLA-A	AGATACACCTGCCATGTGCAGC	GATCACAGCTCCAAGGAGAACC
HLA-B	CTGCTGTGATGTGTAGGAGGAAG	GCTGTGAGAGACACATCAGAGC
HLA-C	GGAGACACAGAAGTACAAGCGC	ACATCCTCTGGAGGGTGTGAGA
HLA-E	CGGCTACTACAATCAGAGCGAG	AATCCTTGCCGTCGTAGGCGAA
GBP1	TAGCAGACTTCTGTTCCTACATCT	CCACTGCTGATGGCATTGACGT
CASP1	GCTGAGGTTGACATCACAGGCA	TGCTGTCAGAGGTCTTGTGCTC
TAP-BP	GAGCCTGTTCTCATCACCATGG	GTAGGCAAAGCTCAAGTCCAGC
TAP1	GCAGTCAACTCCTGGACCACTA	CAAGGTTCCCACTGCTTACAGC
TAP2	ATGCCCTTCACAATAGCAGCGG	CCAAAACTGCGAACGGTCTGCA
PSMB8	CCTTACCTGCTTGGCACCATGT	TTGGAGGCTGCCGACACTGAAA
CD74	AAGCCTGTGAGCAAGATGCGCA	AGCAGGTGCATCACATGGTCCT
B2M	CCACTGAAAAAGATGAGTATGCCT	CCAATCCAAATGCGGCATCTTCA

TABLE S2. HLA Class I genotype of the ovarian cancer cell lines used in Fig. 5, as retrieved from http://celllines.tron-mainz.de [51]

Cell line	A locus	B locus	C locus
A2780	26:03’; 26:03	49:01; 37:04	07:01; 02:02
CAOV3	69:01; 26:01’	49:01; 49:01	07:01; 07:01
OVCAR5	01:01; 02:01	08:01; 44:02	07:01; 05:01
SKOV3	68:01; 03:01	18:01; 35:01	04:01; 05:01
